# Modulating Microbiota as a New Strategy for Breast Cancer Prevention and Treatment

**DOI:** 10.3390/microorganisms10091727

**Published:** 2022-08-27

**Authors:** Huixin Wu, Sebanti Ganguly, Trygve O. Tollefsbol

**Affiliations:** 1 Department of Biology, University of Alabama at Birmingham, 1300 University Boulevard, Birmingham, AL 35294, USA; 2 Integrative Center for Aging Research, University of Alabama Birmingham, 1530 3rd Avenue South, Birmingham, AL 35294, USA; 3 O’Neal Comprehensive Cancer Center, University of Alabama Birmingham, 1802 6th Avenue South, Birmingham, AL 35294, USA; 4 Nutrition Obesity Research Center, University of Alabama Birmingham, 1675 University Boulevard, Birmingham, AL 35294, USA; 5 Comprehensive Diabetes Center, University of Alabama Birmingham, 1825 University Boulevard, Birmingham, AL 35294, USA; 6 University Wide Microbiome Center, University of Alabama Birmingham, 845 19th Street South, Birmingham, AL 35294, USA

**Keywords:** breast cancer, gut microbiota, breast microbiota, probiotics, prebiotics, epigenetics

## Abstract

Breast cancer (BC) is the most common cancer in women in the United States. There has been an increasing incidence and decreasing mortality rate of BC cases over the past several decades. Many risk factors are associated with BC, such as diet, aging, personal and family history, obesity, and some environmental factors. Recent studies have shown that healthy individuals and BC patients have different microbiota composition, indicating that microbiome is a new risk factor for BC. Gut and breast microbiota alterations are associated with BC prognosis. This review will evaluate altered microbiota populations in gut, breast tissue, and milk of BC patients, as well as mechanisms of interactions between microbiota modulation and BC. Probiotics and prebiotics are commercially available dietary supplements to alleviate side-effects of cancer therapies. They also shape the population of human gut microbiome. This review evaluates novel means of modulating microbiota by nutritional treatment with probiotics and prebiotics as emerging and promising strategies for prevention and treatment of BC. The mechanistic role of probiotic and prebiotics partially depend on alterations in estrogen metabolism, systematic immune regulation, and epigenetics regulation.

## 1. Introduction

Breast cancer (BC) affects more than 2 million women each year and became the most frequently diagnosed cancer in the U.S. in 2021. According to recent studies, ‘dysbiosis’, or the alteration in gut or breast tissue microbiota diversity, is frequently associated with BC. While microbiota in breast tissue have direct impact on tumor development, dysbiosis, or reduced alpha diversity of gut bacteria, can also affect tumor development by the production of metabolites that can elicit altered immune response, manipulate estrogen level, or induce epigenetics effects [1,2].

Insoluble dietary fibers, such as cellulose and hemicellulose, are anaerobically fermented by the gut microbiota to produce approximately 500–600 mmols of monocarboxylic acid compounds, such as acetate, propionate, butyrate, and valerate, which comprise the short-chain fatty acid (SCFA) family. A relatively small amount of the SCFAs generated in the lumens of the colon reaches the blood serum after absorption through the mucosa but can exert significant influence in controlling inflammation by modulating activity of immune cells, such as macrophages, natural killer cells, dendritic cells, and T cells [3,4,5]. SCFAs, similar to propionyl- and butyryl- coA, generated by gut microbiota exert their effects in part by inhibiting histone deacetylases (HDACs), thereby elevating acetylation of histone subunits and, subsequently, up-regulating significant genes related to BC development. Recent in vitro studies on colon and breast cancer by Thomas et. al. have brought attention to alterations in activity of histone acetyltransferase (HAT) domains of bivalent proteins (both tumor suppressors and oncoproteins) such as p300, up-regulation of which is a marker for extended cell proliferation and survival and thus poor prognosis [6,7]. 16s ribosomal RNA and shotgun metagenomics followed by a taxonomic analysis reveal that the common species of gut microbiota are Bacteroidetes, Firmicutes, Proteobacteria, Actinobacteria, Verrucomicrobia, and Cyanobacteria [1]. This review aims to elaborate on the specific changes in diversity of gut and breast microbiota and their relationship with the epigenetics of BC. Modulating microbiota by probiotics and prebiotics is becoming a new strategy of BC prevention. This review also discusses the mechanistic roles of probiotics and prebiotics in BC prevention and treatment.

## 2. Methods

Research and review papers identified through PubMed database were searched using key words breast cancer, gut microbiota, breast microbiota, probiotics and breast cancer, and prebiotics and breast cancer. All research and review references were searched from PubMed. Full texts articles were assessed for all references. Publication dates within the past five years were employed. No additional filters were applied. No qualitative or quantitative synthesis was performed. No meta-analysis was performed. Clinical trials were searched in ClinicalTrials.gov using key words microbiota and breast cancer. 

## 3. Gut Microbiota and BC

About $10^{14}$ microorganisms are living in the gastrointestinal track, participating in physiological processes, and interacting with each other and the host [8]. Microorganisms comprise a ‘forgotten organ’ in the human body [9]. The ratio of bacteria cells to human cells is approximately 1:1 [10]. Infants are exposed to maternal vaginal microbes, such as *Prevotella spp*. and *Lactobacillus*, and skin microbes, such as *Staphylococcus* and *Propionibacterium spp*. Breast feeding, consumption of solid food, and changes in hormone levels all contribute to major establishment of gut microbiota [8]. Different bacterial genera colonize at different locations, such as the ileum, stomach, and colon, and actively regulate the intestinal immune system [8]. BC occurrence and development are affected by multiple factors, such as age, hormone level, menopausal stage, inflammation and immunity, and cancer development stage and severity, as well as other factors. Microorganisms in the gut have been shown in previous studies to be involved in the crosstalk between multiple risk factors and BC [2]. The intestinal microbiota population is not only associated with BC stages but also influences clinical outcomes of BC patients. In the early stages of BC treatment, cell autonomous and immune responses are important for cancer development. *Blautia*, *Faecalibacterium prausnitzii*, and *Bifidobacterium* were correlated to clinical stages [11]. For instance, lower abundance of *Blautia sp*. were present in stage 1 compared to stage 3 BC [11]. Safae and colleagues showed that chemotherapy caused over-abundance of gut commensals and had a negative impact on BC prognosis [12]. Dysbiosis is believed to accelerate cancer development by damaging host DNA, producing metabolites to induce inflammation, and causing dysregulation of the host immune system [13,14]. For example, an antibiotic treatment using cephalosporin induced BC and reduced microbiota diversity [15]. An EGFR/Her2 tyrosine kinase inhibitor, lapatinib, can induce diarrhea, decrease microbiota delivery, and, possibly, lead to dysbiosis [16]. Besides some negative impacts of pathogenic bacteria, however, microbiota populations can catalyze chemical reactions to accelerate anti-cancer drug biotransformation [17].

### 3.1. Gut Microbiota Composition Is Different in BC Patients and Subtypes of BC

Patients with breast carcinoma and those with benign tumors had different gut microbiota composition. Peidong et al., found that *Citrobacter* was significantly higher in a malignant tumor group compared to a benign tumor group. The KEGG pathway analysis revealed increased glycan biosynthesis and metabolism, and lipopolysaccharide biosynthesis in the malignant tumor group compared to the benign tumor group [18]. Further, early-stage BC patients have different gut microbiota composition compared to heathy controls. Alpha diversity Shannon index was lower in BC groups in which fecal samples were collected from newly diagnosed patients before any treatment. Moreover, there was a higher abundance of Firmicutes and *Verrucomicrobiaceae* and lower abundance of Bacteroidetes. Moreover, the relative abundance of *Odoribacter* sp. and *Butyricimonas* sp. was observed among BC patients [19]. In addition to differences of gut microbiome observed within early-stage BC cases, patients undergoing neoadjuvant chemotherapy have different fecal metabolic profiles due to altered microbiota population. Lactate and fumaric acid were down-regulated in chemotherapy groups compared to patients before treatment, as shown by Zidi et al. [19]. SCFAs, including propionate, butyrate, and acetate, increased in patients who received three cycles of chemotherapy [19]. Another study focused on intestinal microbiota in early BC treatment showed microbiome is associated with prognosis of multiple types of early BC [12]. The abundance of Streptococcus, Lachnospiraceae, etc. were higher in aggressive tumors, while the abundance of certain health-related commensals was higher in less aggressive tumors. More importantly, distinct bacterial species were identified with respect to prognosis to be associated with the cancer status or healthy status. Commensal bacteria could be promising for characterizing BC patients with better or worse prognosis in future analysis. Furthermore, side-effects induced by chemotherapy were associated with decreased beta diversity of fecal microbiota [12]. Overall, these results indicate fecal metabolites potentially change correlating with the treatment period, and gut microbiome can change metabolic profiles to affect BC risk.

Gut microbiota has also been associated with emotion stress treatment efficiency in BC patients. Chemotherapies are known to decrease cancer recurrence and alter gut microbiota in BC survivors. With emerging evidence indicating the microbiota–gut–brain axis affects anxiety disorder, Ryo et al., found that among women diagnosed with invasive BC, relative abundance of Bacteroides genus was directly and significantly associated with fear of cancer recurrence (FCR). Higher FCR was associated with lower microbial diversity where higher relative abundance of *Bacteroides* and lower relative abundance of *Lanchnospiraceae* and *Ruminococcus* were identified [20]. In Her2-negative BC patients treated with metronomic apecitabine, higher abundance of *Slackia* was associated with lower progression-free survival (PFS), while higher abundance of *Blautia obeum* was associated with higher PFS [21]. These studies shed light on gut microbiota and pathophysiology which, potentially, can affect prognosis of BC treatment.

### 3.2. Gut Microbiota Dysbiosis and the Relationship between Microbiota and the Host in BC

BC incidence has been shown to relate to microbiota dysbiosis in the past decade. *Methylobacterium radiotolerans* and *Sphingomonas yanoikuyae* were enriched in breast tumor tissue and paired normal tissue, respectively [22]. Breast tumor tissue of estrogen receptor-positive BC is colonized by *Methylobacterium* [22]. *Sphigomonas* was shown to activate Toll-like receptor 5 (TLR5) that inhibits BC development [23]. Reduced total bacterial DNA load as well as lowered basal level of antibacterial response gene indicated that a lower microbiota population in tumor tissue was related to limited immune response in the host [22]. Rosean et al., demonstrated that disturbing microbiota homeostasis by antibiotics to form a pre-established commensal dysbiosis can result in severe tissue inflammation, enhanced fibrosis, and tumor cell dissemination in a hormone receptor-positive BC mouse model [24]. McKee et al., also showed disturbing gut microbiota resulted in aggravated breast tumor growth [25]. An antibiotics cocktail eliminated some bacterial species and resulted in significantly accelerated tumor growth in the BrCa mouse model which harbors mutations of BRCA genes that repair damaged DNA. Furthermore, SCFAs, such as butyrate and propionate, as well as medium-chain fatty acids significantly decreased after antibiotics treatment. However, two strategies rescued this effect; *Faecalibaculum rodentium*, or mast cells, can both rescue this negative influence of antibiotics [25]. Therefore, maintaining a homeostasis environment between bacteria and host is critical for optimizing the beneficial effects that humans can obtain from microorganisms. Dysbiosis should be considered carefully to prevent pathobionts from exacerbating the disease while modulating gut microbiota is becoming a promising strategy for enhancing therapeutic effects in the future.

While gut microbiota is shaped by hosts, altered microbiota can have positive or negative influences in hosts. For example, enteric bacteria *Helicobacter hepaticus*-infected mice can significantly enhance the development of mammary tumors by up-regulating the TNF-α pro-inflammatory cytokine [26]. A subset of interleukin-10 competent regulatory T cells rescued this negative effect of *H. hapaticus*, suggesting that the human immune system plays important roles in suppressing negative influences from some gut bacteria strains [26]. Consuming a high fat diet after colonization of *Erysipelotrichaceae* increased inflammation in the gut [27]. Individuals are benefiting from a positive influence from gut microbiota as it is not only affecting estrogen levels but also producing metabolites that have anti-cancer potentials. Lithocholic acid decreased BC cell proliferation and inhibited epithelial to mesenchymal transition in vitro [28,29]. Lactate and SCFAs, such as butyrate, propionate, and acetate, are generated by bacteria after indigestible carbohydrate fermentation [29] (Table 1). 

SCFA producers not only provide support for the host, but also maintain a viable environment for other bacteria that produce indole and hydrogen sulfide [46]. Butyrate is known for its anti-tumor and anti-inflammation effect. Bacteria producing butyrate include *F. prausnitzii*, *Roseburia intestinalis*, and *Eubacterium rectale* [28]. Acting as an HDAC inhibitor, sodium butyrate has shown promising anti-triple negative BC (TNBC) impact both alone and in combination with anti-cancer agents [47]. The amino acid, cadaverine, is also known for its anti-tumor effect. Cadaverine inhibited BC cellular growth by reducing migration and invasion of cells, as well as suppressing epithelial to mesenchymal transition [28]. Bacteria secrete metabolites and various compounds, as well as extracellular vesicles to the extracellular environment. Extracellular vesicles are lipid layer-enclosed cytosol released by bacteria for communication between cells and transporting content from one cell to another. Previous investigators added extracellular vesicles produced from *Klebsiella pneumoniae* to the MCF-7 estrogen receptor-positive BC cell line under tam oxifen treatment. Extracellular vesicles decreased expression of CCNE1 and p-ERK, enhancing the anti-tumor effect of tamoxifen [48]. On the other hand, gut microbiota can have a negative impact on the host. Martina et al., showed that gut microbiota can weaken the efficacy of trastuzumab in Her2-positive BC. Trastuzumab attracts immune cells to tumors that over-express Her2 on their cell surface. Its antitumor activity is diminished by antibiotic treatment which lowered the abundance of *Lachnospiraceae*, *Turicibacteraceae*, *Bifidobacteriaceae*, and *Prevotellaceae*. Antibiotic treatments that cause dysbiosis decreased CD4^+^ T cells and granzyme B+ cells, reduced dendritic cell activation, and inhibited the release of interleukin 12p70 [49].

### 3.3. Clinical Studies on Gut Microbiota in BC

Studying microbiota in BC has translational and clinical significance as the breast microbiota colonize normal and cancerous breast tissue and the gut microbiota and their metabolites are closely related to host immune responses and have been associated with BC progression. Various factors, such as BC stages, subtypes of BC, and therapies, including neoadjuvant therapy, chemotherapies, and antibiotics treatments, can affect the thera peutic outcome as well as microbiota composition (NCT03885648). In vitro and in vivo studies of gut and breast microbiota in BC have been actively investigated during the past two decades. Clinical trials are needed for understanding the association of microbiota composition with the risk of BC in humans and to bring scientific research to publicly available supplements and treatments for BC patients. Completed clinical studies have explored microbiome in BC patients, such as the influence of antibiotics in surgery and the association of intestinal bacteria with BC risks, as well as probiotics and breast health (NCT03702868, NCT01461070, and NCT03290651) [50]. Several ongoing clinical trials focus on the impact of drugs, diet, and probiotics on gut microbiota in BC patients undergoing chemotherapy treatments. The abundance of *Akkermansia*, a promising probiotics candidate, has been evaluated in a presurgical weight-loss trail of overweight and obese BC women. Microbiome alpha-diversity and the abundance of *Akkermansia* decreased while pro-inflammatory marker interleukin-6 increased [51]. Another innovative clinical trial explored the immune-boosting function of probiotics in BC. An over-the-counter probiotic delivered to stage I-III BC patients three times a day increased CD8^+^ T cells a month after baseline indicating probiotics induced protective immune response against pathogens as well as tumor surveillance (NCT03358511). In a case-control clinical study, BC women at early stages who underwent surgical intervention were involved. Both gut microbiota and breast microbiota will be assessed and correlated to BC risks (NCT03885648). Evaluating gut and breast microbiota in the same individuals is critical for establishing association between microbiome and BC systematically. Since the efficacy of neoadjuvant therapies can be influenced by the microbiota in different individuals, clinical trials involving BC patients will improve the application of probiotics in supplementary therapies and neoadjuvant therapies

### 3.4. The Mechanistic Role of Breast Microbiota in BC

Though researchers have found a strong correlation between microbiome and BC at different stages and ages, the mechanism of action is in need of further study. Firmicutes and Bacteroidetes are the most prevalent phyla found in feces in early-stage BC cancer patients. Stage II and III cancer patients had significantly more Bacteroidetes, *Clostridium coccoides*, *Clostridium leptum*, *F. prausnitzii*, and *Blautia* spp. compared to stage 0 and I patients. Moreover, *Blautia* spp. increased as the prognostic grades increased [11]. Sheetal et al., investigated a pro-carcinogenic colon microbe *Bacteroides fragilis* that promotes breast tumorigenesis and metastasis. Mammary gland or gut colonized with enterotoxigenic *B. fragilis* induced mammary gland epithelial cell proliferation. Toxins released by *B. fragilis* increased metastasis and invasion in BC cell lines and xenografts. This biological effect in vitro was mediated by toxin activated Notch1 and beta-catenin axes [52]. Regulating tumor infiltrating lymphocyte (TIL) is another means by which gut microbiota can influence BC. Shi et al., classified TIL as three groups based on the proportion of filtrated area in tumor and adjacent areas. High TIL individuals had better chemotherapy outcomes and had statistically different beta diversity distribution of gut microbiota compared to the low TIL group. Higher abundance of *Methanosphaera* and *Anaerobiospirillum* and lower abundance of *Mycobacterium*, *Rhodococcus*, etc., were identified in the high TIL group compared to the low TIL group [53]. Moreover, species *Barnesiae*, that is known to regulate estrogen metabolism, had higher abundance in the low TIL group, indicating *Barnesiae* may be a potential risk factor promoting cancer development in low TIL cases [53]. TLRs respond to pathogen-associated molecular patterns to induce the production of cytokines and chemokines [54]. For example, TLR2 expression is higher in MDA-MB-231 compared to less aggressive MCF-7 BC cells. The mechanism of higher aggressiveness seen in the MDA-MB-231 cell line is partially mediated by induced NF-kB, interlukin-6, and MMP9 levels [55]. These results suggest that immunity is closely related to carcinogenesis. Since gut microbiota modulate both lymphocytes and neutrophils, immune-related responses to gut microbiota can have a great impact on mammary carcinogenesis. Lakritz et al., showed neutrophiles-depleted mice were more susceptible to *H. hepaticus*-triggered tumor development [56,57]. Decreasing proinflammatory cytokines and inflammation can prevent tumor development during the early stages of BC [54]. Furthermore, effector CD8^+^ T cells inhibited the growth of Her-2/neu tumor cells in anti-cancer immunotherapy. Calories intake is another factor affecting BC treatment outcome. Restricting calories intake during TNBC radiotherapy treatment greatly decreased tumor development in mice compared to normal diet supplying a normal amount of calories. The mechanism was mediated by down-regulation of a well-known metastasis-driving pathway, insulin-like growth factor, and Akt pathway [58,59].

Hormone metabolism is another important way for gut microbiota to affect their host, while the microbiota is, in turn, affected by host menopausal stage and hormone levels. Pre- and post-menopausal status is known to have significantly different hormone levels. Ming-Feng et al., showed alpha-diversity significantly decreased in premenopausal BC patients compared to control. *Bacteroides fragilis* was found in premenopausal individuals, while *Klebsiella pneumoniae* was found in aged post-menopausal individuals [60]. In studies focused on post-menopausal cases, Jia et al., found BC patients had higher microbial diversity compared to controls, although the diversity has been found to decrease in BC patients in most cases. Within multiple species that were enriched in cancer patients, some species were positively correlated with estradiol levels and negatively correlated with CD3^+^ CD8^+^ T cells [61]. Moreover, gut metagenome showed enriched genes participating in lipopolysaccharide biosynthesis and the carbohydrate phosphotransferase system in cancer patients [61]. Post-menopausal BC patients harbor significantly reduced alpha diversity especially in IgA-coated and Ig-A noncoated taxa. Species belonging to IgA-coated Proteobacteria and Firmicutes, and genus belonging to IgA-noncoated Firmicutes, such as *Ruminococcus* and *Clostridium*, are associated with BC cases [62]. According to these previous studies, the difference in gut microbiota composition is more distinct comparing post-menopausal BC patients to controls. 

Steroid hormones play important roles in BC. For example, in gastrointestinal lumen, conjugated estrogen is deconjugated by beta-glucuronidase bacteria that supply free estrogen to breast tissue through the circulatory system [63]. A higher estrogen level has been linked to increased risk of BC. Estrogen level is affected by intestinal microbiota because they produce beta-glucuronidase enzymes that deconjugate estrogen metabolites. Estrogen metabolites can remain in the circulation to raise the estrogen level. Gut microbial beta-glucuronidase and estrogen level are regulated bidirectionally. Beta-glucuronidase is structurally specific for reactivating estrogen. Ervin et al., confirmed in vitro that certain members in beta-glucuronidase enzymes with distinct structure can reactivate estrogen glucuronidases [64]. Estrogen level is also affected by bacterial estrobolome in host intestine that increases the risk of estrogen-receptor positive BC among post-menopausal patients [65]. Decreasing diversity of bacteria and dysbiosis of gut microbiome resulted in excreted estrogen and higher risk of BC [66]. Bacteria that produce beta-glucuronidase include *Clostridia*, *Ruminococcaceae*, and *Eschherichia*. Potential beta-glucuronidase inhibitors include the probiotics *Bifidobacterium longum*, flavonoids, and silybin, as well as others [67]. The relative abundance of *B. longum* positively correlated with docosahexaenoic acid, an essential nutrient that has beneficial effects in BC survivors [30]. This study reinforced the important relationship among gut microbiota, metabolites, and BC prognosis. Estrogen level can also be manipulated by dietary fiber consumption and gut microbiota digestion [68]. In addition to circulating estrogen levels, hormone receptor level is another important factor in BC development. A pilot study that explored the association between gut microbiome and BC risk factors, including receptor status, stage, and grade of BC, showed that women with Her2+ BC had 12–23% lower alpha diversity, higher abundance of Bacteroidetes and lower abundance of Firmicutes compared to Her2- patients [69]. On the other hand, gut microbiota can also have a positive impact on estrogen signaling. Intestinal microbes can break down polyphenols and plant-derived phytochemical compounds to form estrogen-like compounds [70]. Phytoestrogens and plant-derived xenoestrogens compete with estrogen for its receptor; therefore, they can act as anti- estrogen agents [71]. Bacteria ferment plant lignans into enterodiol and enterolactone which was shown to serve as an anti-estrogen agent that can lower the risk of BC [72,73]. Furthermore, gut microbiota can modulate estrogen metabolism. The ratio of estrogen metabolites, such as estradiol and hydroxylated estrogen, to parent estrogen are positively related to higher risk of BC. This effect has been evaluated on post-menopausal BC patients with higher levels of circulating estrogen [74,75]. These studies indicate that bacteria that have positive or negative impacts on BC partially depended on their roles in estrogen metabolism for these effects.

Among several factors that significantly alter gut microbiota in BC, such as diet, obesity, alcohol consumption, hormone levels, and antibiotic treatment, diet and gut microbiota are closely related because the presence of bacteria is constantly modulated by our daily dietary patterns [76]. A diet rich in red meat and animal fat has been associated with increased risk of BC. Among post-menopausal patients, western diets had a negative impact on hormone receptor-positive patients. However, the significant effect of a healthy diet (i.e., vegetable, fruit, and fish) was only significant among premenopausal patients, indicating that diet may greatly affect the health of individuals at younger ages [77]. Furthermore, diet is digested by a large number of bacteria called degraders through cross-feeding. Fibers and polysaccharides are converted to oligosaccharides and SCFAs by primary degraders, such as *B. thetaiotaomicron*. These nutrients are further degraded to monosaccharides and other downstream products by secondary degraders, such as *Eubacterium rectale* [78]. Gut bacteria have been shown to participate in food digestion as well as phytochemical breakdown. For example, *Flavonifractor plautii* can convert catechin and epicatechin to velaric acids, and *Slackia equolifaciens* can turn soy isoflavones to equol and *O*-desmethylangolensin [79]. The exchange of nutrients and chemicals between bacteria and host occur consistently rendering it extremely important to study gut microbiota as a ‘new organ system’ in the human body. 

## 4. Breast Microbiota and BC

The breast is a complex and favorable microenvironment composed of host tissue, epithelium and stroma, and microorganisms [80]. Breast tissue and ducts are not sterile. Instead, they are colonized with a diverse community of microorganisms originating from breast skin, nipples, mammary areolas, and entero-mammary pathways where the same genera of butyrate-producing bacteria have been found in both maternal feces and milk [73,81,82]. Bacteria can be transported to other areas by dendritic cells [83]. For example, CD18^+^ cells and dendritic cells can transport gut microbiota to lactating breast tissue [84]. Bacteria can also travel to organs such as mesenteric lymph nodes and liver [85]. Infants consume $10^{4}$ to $10^{6}$ commensal bacteria on average per day [86]. The ‘core’ breast bacteriome consists of *Staphylococcus*, *Streptococcus*, *Lactobacillus*, and *Propionibacterium* to main tain the homeostasis in the breast microenvironment as summarized by Okunola et al. [87]. Being a distinct community of microbiota different from that found in other parts of the body, breast microbiota interacts with the host by immune modulation and the production of enzymes and metabolites, such as beta-glucuronidase, SCFAs, lipopolysaccharide (LPS), bile acids, etc. [88]. In mouse mammary tumor, *Butyrivibrio fibrisolvens*-related species in Firmicutes phylum were found in tumors of a xenograft mouse model [89]. In human mammary tissue, diverse populations of bacteria were found in human mammary tissue with and without BC, including the main phylum Proteobacteria, followed by *Enterobacteriaceae*, *Staphylococcus*, *Propionibacterium*, and others [90]. Although the major phylum and taxa of bacteria identified in breast tissue were different from microbiota identified in the GI tract, bacteria established in these two distant located areas can affect each other through metabolomes and immunity.

### 4.1. The Origination of Breast Microbiota

Bacteria in breast tissue is hypothesized to originate from translocation from the gastrointestinal tract, nipple–areolar complex, or oral contact. Bacterial composition in breast tissue is distinctly different from gut microbiota. Major microorganisms in the gastrointestinal track include Bacteroidetes, Firmicutes, Proteobacteria, and Actinobacteria [91]. Different from that in the GI track, major microorganisms in breast tissue are *Staphylococcus*, *Serratia*, and *Streptococcus*, among others [73]. Despite differences in microbiota composition in gut and breast tissue, fecal transplantation showed changing gut microbiota can lead to alteration in breast microbiota [92]. Transplanting control mice with fecal samples from mice fed high-fat diet (HFD) recapitulated tumorigenic effects in mice on high-fat diet. Transplanting HFD mice with fecal samples from control mice resulted in reduced tight junction disrupting bacterial lipopolysaccharide in HFD mice [92]. These results suggest breast microbiota composition was influenced by gut microbiota.

### 4.2. Breast Microbiota Composition Is Different in BC Patients and Subtypes of BC

The microbiome of pathogenic tissue is also greatly different from healthy tissue. Thompson et al., showed that Proteobacteria, Actinobacteria, and Firmicutes are major phyla in breast tissues. The relative abundance of these phyla is different in breast tissue and adjacent normal tissue. Proteobacteria was more prevalent in breast tumors while Actinobacteria was more prevalent in normal tissue [93]. When microbiome is analyzed in the same individual, microbiome composition of BC tumor tissue harbored lower richness of bacteria compared to healthy adjacent tissue [94]. The relative abundance of Actinobacteria and *Propionibacteriaceae* were lower in tumor tissue, while the relative abundance of Proteobacteria, Firmicutes, and Alphaproteobacteria was higher [94]. Comparison of breast microbiota between women that have BC and women with benign breast disease showed correlation of bacterial genera with malignancy. Higher abundance of *Fusobacterium*, *Atopobium*, and *Gluconacetobacter*, as well as others, were correlated with malignancy [80]. Moreover, breast tissue microbiome changed at different stages of BC as evaluated by a clinical trial [73]. As the BC stage progresses, changes in microbiota can subsequently down-regulate biosynthesis of flavonoids while up-regulating biological process related to glycerophospholipids and ribosomes [95]. Moreover, Urbanaik et al., also found that compared to normal breast tissue collected from healthy individuals, BC patients had higher relative abundance of Bacillus, Enterobacteriaceae, and Staphylococcus [96]. These three genera were also identified in normal breast tissue in previous research, indicating the higher concentration, instead of the presence, of certain bacteria is associated with breast cancer incidence. Moreover, *E. coli* and *Staphylococcus epidermidis*, but not *Bacillus* or *Streptococcus*, can produce colibactin that causes DNA double-strand breaks (43, 29). This is hypothesized to promote BC development.

In all subtypes of BC, *Mobiluncus*, *Brevundimonas*, and *Actinomyces* were presented at higher abundance [97]. Her2 type of BC had higher abundance of *Akkermansia* [98]. A higher level of fungal species was identified in estrogen receptor-negative BC compared to other types of BC. TNBC was associated with a more distinct microorganism population [99]. The presence of virus, bacteria, and parasite were different in TNBC breast tissue compared to the control. The relative abundance of *Arcanobacterium* as well as *Brevundimonas*, *Sphingobacteria*, and *Providencia* was higher in breast tissue [99]. The majority of studies on breast microbiota were performed by 16S rRNA sequencing and/or metagenomic sequencing. Culturing bacteria from the breast tissue and tumor are more useful for studying characteristics of specific bacterial strains. A study that compared culturable bacteria observed in breast tumor and benign lesions showed the BC group had increased cultivated *E. coli* and *S. aureus* and no bacteria was cultivated in the benign lesions group [100]. Moreover, samples collected from senior women had more culturable bacteria than women at younger ages [100]. In addition to dominant cultivatable bacteria, Bifidobacterial and lactic acid-producing Lactobacillus were also identified in the milk of healthy women [101]. Overall, these results show that the prevalence of distinct types of microorganism are higher in BC tissue compared to healthy tissue. Microorganisms might be used as targets for evaluating prognosis of BC.

### 4.3. Breast Milk Microbiota

Breast milk is also a non-sterile environment harboring viruses, fungi, and bacteria. Milk is hypothesized to contribute to fungi and bacteria establishment in the infant gut [81]. Dysbiosis of maternal milk microbiota can be passed on to infants and, potentially, result in alteration of gut microbiota in infants [81]. Specific microbiota was identified in breast milk. Chan et al., identified major flora of microbiota in nipple aspirate fluid (NAF) [23]. Compared to healthy controls that had higher abundance of *Sphingomonadaceae*, BC survivors had higher abundance of *Alistipes* in the ductal area. Interestingly, higher beta-glucuronidase activity was associated with microbes identified in BC samples [23]. Microbiota composition was different in breast tissue and NAF. NAF is an important means of exposing infants to a variety of microorganisms. Therefore, studying and understanding microbiota in the breast milk of healthy nursing mothers and breastfed infants are important as beneficial bacteria can help maintain a healthy status of hosts and infants in the later stages of life [102]. Infants are exposed to bacteria in maternal milk where bacteria originate [103]. Babakobi et al., showed postpartum maternal diet affects maternal milk microbiota and infant gut microbiota composition [104]. In breast milk, the abundance of *Streptococcus* was negatively related to dietary consumption of unsaturated fatty acids, folic acid, and vitamin B-12. *Staphylococcus hominis* correlated to medium-chain saturated fatty acids. Though bacterial growth depends on oxygen requirement, they found *Streptococcus*, *Escherichia*, and *Staphylococcus* in stool samples as well as milk samples [104]. Therefore, diet intake can cause microbiota changes in both gut and maternal milk which can further modulate infant gut microbiota. Compounds in milk, such as fucosyltransferase 8 (Fut8), were also studied for their impact on infant gut microbiota [105]. Catalyzing core fucosylation in milk, Fut8 selectively promoted the growth of *Bifidobacterium* spp. and *Lactobacillus* spp., because *Bifidobacterium* can metabolize human milk oligosaccharides fucosyllactose in breastfed infants [102]. Moreover, B lymphocytes are activated by metabolites produced during catalysis. Furthermore, alpha-diversity and beta-diversity of infant gut microbiota were influenced by nursing mothers [104]. *Streptococcaceae* can be directly passed to infants by breast milk [104]. The impact of the nursing mother on infants during lactation and early development might be important at later life stages. Overall, breast milk promotes growth of probiotics and induced protective immune response in infants [105].

Breastfed infants gain a complex population of bacteria, viruses, and fungi from the mother based on the evidence that mammary milk microbiota is dominant in infant fecal microbiota [106]. Breast milk has anti-pathogen and anti-cancer effects because it contains antimicrobial compounds, such as organic acids and hydrogen peroxide. Consuming milk can also strengthen the infant gut barrier by decreasing intestinal permeability. Several bacterial species served as immune cells activators during immune programming of infants. Modulating milk microbiota by diets and/or supplements may be used to shape the microbiota toward a protective composition of bacteria that potentially benefit both the mammary gland and infants. Metabolites discovered in human milk are relevant to health of the mammary gland and infant. Functioning as prebiotics, human milk oligosaccharides (HMOs) were found in milk that contained *Bifidobacterium* and *Staphylococcus* [107]. Furthermore, two butyrate-producing bacteria, *Roseburia* and *Eubacterium*, were found to grow in the presence of HMOs [108]. Among a variety of metabolites identified, SCFAs are produced by microbiota and play important roles in host-immune and neuroendocrine systems [109]. SCFAs have also been associated with anti-cancer effects by previous studies (Table 1). SCFAs levels increased in six-month-old infants during the postpartum period who received Lactobacillus isolated from mammary milk, indicating probiotics isolated in milk can be used to shape gut microbiota in infants toward a protective microbiota composition [110]. Another bacteria species of Lactobacillus can also exert probiotics effect. This species had immune-modulatory DNA motifs and open reading frames to regulate host immunity, as well as nitrogen metabolism and stress response in the GI track [111,112]. A probiotics cocktail administered orally to pregnant women created higher abundance of *Bifidobacterium* and *Lactobacillus* in milk microbiome compared to the placebo group [113]. However, this effect was not seen when administering milk containing bacterial strains to pregnant women.

### 4.4. The Mechanistic Role of Breast Microbiota in BC

The mechanistic role of breast microbiota in BC is mediated by hormone level regulation, inflammation and immunity, dietary patterns, and the bacterial metabolome. Estrogen level plays an important role in BC development, and it is bidirectionally regulated by beta-glucuronidase level in the gut. Interestingly, previous studies found that compared to healthy controls, beta-glucuronidase level was higher in nipple aspirate fluid in BC patients [114]. Beta-glucuronidase is utilized by bacteria and promotes the growth of certain bacteria strains. High-fat diet not rich in fibers was shown to increase beta-glucuronidase activity. Alcohol intake and adiposity can also increase beta-glucuronidase level [115]. Therefore, HFD can increase beta-glucuronidase level to regulate estrogen level which is positively correlated to higher BC risk. BC risk is positively correlated with chronic inflammation status. For example, increased cyclooxygenase 2 and prostaglandin E2 can induce the expression of aromatase and estrogen that promote BC [116]. There have been emerging needs to study the mechanistic role of breast microbiota in BC [117]. Banerjee et al., showed Proteobacteria is the predominant phylum in both breast tumor and normal tissues. In addition, normal adjacent tissue had increased the abundance of Actinobacteria [99]. However, the mechanistic role of breast microbiota in BC needs further exploration. Banerjee eta al. proposed epithelial-to-mesenchymal transition (EMT) was associated with higher abundance of *Listeria fleischmannii*. Genome-regulating cell cycle checkpoints and mitosis were associated with *Haemophilus influenzae* [99]. An anti-tumor role of bacterial metabolite was mediated by suppressing EMT and metastasis and reducing cell stemness [118]. Diet can affect microbiota in both breast tissue and the GI tract. In addition to regulating circulatory estrogen levels, diet alone can shape breast microbiota. The Mediterranean diet significantly increased relative abundance of mammary gland *Lactobacillus* which was present at lower abundance in tumors compared to benign tissue, indicating the importance of diet pattern in protective breast microbiota formation [119]. Gut microbiota also plays an important role in modulating breast microbiota. The gut environment can secrete compounds to affect epithelial-to-mesenchymal transition. For example, secondary bile acids that are exclusively secreted by bacteria can inhibit BC cellular proliferation by down-regulating several intermediates in the Krebs cycle and oxidative phosphorylation pathways [120]. The serum level of lithocholic acid, a secondary bile acid, was lower in BC patients compared to healthy control, indicating the protective role of secondary bile acid and other bacterial metabolites in preventing BC.

### 4.5. Modifying Breast Microbiota in BC

Modifying breast microorganisms is closely related to BC risk. Diet has been studied and compared to FDA-approved drugs regarding their ability to modify the breast microenvironment. Dietary flaxseed affected microenvironment in normal breast tissue of post-menopausal women by increasing several interleukin levels after treatment, and the mechanism was similar to that seen in tamoxifen treatment. Flaxseed and tamoxifen also regulated other different types of interleukins. Therefore, in addition to FDA-approved drugs, dietary treatment can also stimulate inflammatory response by regulating different factors [121]. Breast implant is another main reason of microbiota modulation in breast tissue. Gram-negative bacteria *Pseudomonas aeruginosa* secrete pyocyanin to escape the host immune system to cause early infection in breast implants [122]. Necrotizing soft tissue infection is also caused by bacterial infection [97]. *P. aeruginosa* can also play an important role in chemotherapy as it greatly increases after chemotherapy. However, high concentration, not low concentration, of *P. aeruginosa* inhibited the growth of multiple BC cell lines [123]. Moreover, this particular bacteria species can enhance the effect of doxorubicin mediated by increasing production of *Pseudomonas* quinolone signal that inhibited NF-kB pathways as well as iron levels to suppress BC development [123].

**Figure 1:**
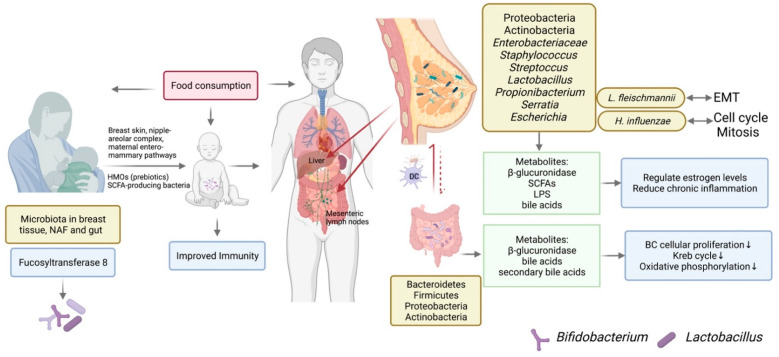
The origin and anti-cancer roles of breast microbiota and milk microbiota. NAF, nipple aspirate fluid; HMOs, human milk oligosaccharides; DC, dendritic cell; SCFAs, short-chain fatty acids; LPS, lipopolysaccharides; and EMT, epithelial–mesenchymal transition.

Breast microbiota affects host immune response mediated by pattern recognition receptors in the tumor microenvironment [124]. The gut–breast microbiota axis can also shape the systemic immunity and regulate estrogen and bile acid levels [124]. Studying breast microbiota is translationally important. Overall, there are many factors modulating breast microbiota composition in different cases (Figure 1). Precision and personalized medicine or dietary supplements may be promising fields for treating breast dysbiosis in the future. When targeting breast microbiota for prognosis and BC treatment, multiple factors need to be considered, including dietary habits, and toxicity and immunity in the breast tumor microenvironment.

## 5. Modulating Microbiota by Probiotics and Prebiotics in BC

Gut microbiota is closely related to BC development and progression and is correlated to BC stages. Probiotics can maintain microbiota homeostasis, outcompete harmful bacteria, and produce beneficial metabolites. Modulating gut microbiota composition by consuming probiotics or prebiotics that stimulate the growth of probiotics has been shown to attenuate side-effects such as dysbiosis of chemotherapy treatment clinically and slow down the development of BC. For example, probiotics supplementation can reduce the incidence of BC chemotherapy-related cognitive impairment mediated by metabolite mentha-1,8-dien-7-ol [50]. Some probiotic bacterial species, such as *Clostridia* and *Bifido bac teria*, can also be genetically modified to express tumor suppressor genes, suicide genes, and tumor-associated antigens, making them more constructive and profitable in anti-tumor treatments [125].

### 5.1. Probiotics in BC Preventions

Bacteria populations established in the host share the environment and nutrients and compete with other bacteria to survive. The growth of one bacterial species can be affected by the presence of others. For example, *H. pylori* caused less acute gastrointestinal pathology when co-colonized with *Lactobacillus murinus* and *Clostridium* [126]. Beneficial bacteria compete with pathogenic bacteria to reach a homeostasis state in the human body. Probiotics are bacteria that help maintain a homeostasis state and prevent dysbiosis. Probiotics are becoming a promising supplementary treatment for BC patients under chemotherapy treatment. The condition of the gastrointestinal tract in patients is greatly improved by consuming prescribed dosages of probiotics. During the past decade, probiotic treatment has stimulated increasing attention and is more widely used in clinical cases and research fields. Probiotics such as *Bifidobacterium* suppress the growth of pathobionts, preventing a dysbiosis condition in the GI tract, and produces beneficial metabolites that are transported by the circulatory system to distant organs in the body. A mixed probiotic (*Bifidobacterium longum* BB536 and *Lactobacillus rhamnosus* HN001) administered to BC survivors increased microbiota diversity and significantly decreased the ratio of Bacteroidetes to Firmicutes. A probiotic supplement also decreased body mass index and insulin resistance [127]. Probiotics can be ingested from fermented food or as commercially available capsules. Modulating microbiota composition is becoming a promising strategy for inhibiting carcinogenesis. Previous study has shown that breast milk-derived *Lactobacillus rhamnosus* ameliorated inflammation and tumorigenesis in a colitis-associated colorectal mouse model [128]. Considering the protective effect of probiotics and the strong association between probiotics treatment and alleviated BC patient cases, questions have been raised regarding the mechanistic roles of probiotics in BC prevention.

#### 5.1.1. Mechanistic Roles of Probiotics in BC

Mechanistic roles of probiotic bacteria species have been studied during the past decade. *Bifidobacterium* and *Lactobacillus* enhance immune response to eliminate tumor cells in tumor microenvironment [129]. A well-known probiotic, *Lactobacillus reuteri*, was found by Lakritz et al., to have anti-cancer influence in mouse models. [130]. *L. reuteri* rescued increased risk of mammary tumor in Swiss mice on a westernized diet. Oral administration of *L. reuteri* in Her2 mutant mice inhibited tumor development. Its anti-tumor function was mediated by induced CD4^+^ CD25^+^ lymphocytes which elicit transplantable protection in mice [130]. Another probiotics strain, *Lactobacillus acidophilus*, orally delivered to BALB/c mice significantly decreased tumor growth rate by inducing the production of interlukin-12 [131]. Mice fed with *Lactobaciilus helveticus*-fermented milk had elevated IgA and CD4^+^ cells in their mammary glands, and increased interlukin-10 [132]. These results indicate the anti-cancer effect of probiotic strains is mediated by host immunity.

Probiotics and immunotherapies are closely related. Bacterial species have been known to have an important role in immunotherapy, and their mechanisms of action are critical for improving immunotherapies. The population of *Sphingomonas* was significantly reduced in non-effective anti-programmed death receptor therapy because of increased inflammation and reduced CD8^+^ T cells [133,134]. However, probiotics *Bifidobacterium* can rescue this situation by inducing the production of interferon-gamma, CD8^+^ tumor-specific T cells and dendritic cells and facilitate anti-PD-L1 immunotherapy [135]. Selected bacterial species, including *Enterococcus*, *Ruminococcaceae*, and *Akkermansia*, can increase the efficacy of PD-1 blockade treatment and with *Enterococci* are being studied as a new probiotics candidate [136]. A BC patient who reacted to PD-1 checkpoint inhibitor had higher abundance of *Akkermansia muciniphila*. Fecal transplant from patients to recipient mice rendered a good response to PD-1 blockade in recipients, confirming the importance of protective bacterial colonization in immunotherapy [137]. Another commensal bacterium, *Bacteroides fragilis*, as a probiotics candidate, can activate Th1 cells to enhance anti-cytotoxic T lymphocyte-associated protein 4 [138]. Based on previous research on TLR agonists as vaccine adjuvants for anti-cancer therapies, Singh et al., found BC mice exposed to TLR4 and LPS had a better outcome than TLR4- or microbial-deficient mice [139].

In addition to regulating hormone levels and host immune responses, gut microbiota can also affect distant organs by producing metabolites that travel in the circulating system. Investigators are seeking additional approaches to prevent BC using probiotic supplementations to understand probiotic metabolite profiles and their mechanistic roles as well as their clinical significance.

#### 5.1.2. The BC Prevention Roles of Probiotics and Their Metabolites Are Investigated In Vitro

Probiotics are primarily shown to increase SCFA production with a concurrent decrease of pathobionts. Many prevention studies were performed to associate fecal and serum fatty acid production with an elevated population of probiotics in gut microbiota. Several members in the SCFA family are well-known HDAC inhibitors that are involved in epigenetics regulations, cell proliferation, immunity responses, programmed cell death, and other processes [43]. Being produced by two major phyla of bacteria, propionate and acetate are primarily produced by Bacteroidetes, and butyrate is produced by Firmicutes [43]. In a study on BC, SCFAs were found to bind to G-protein coupled receptors that induce signaling cascade and phosphorylation of MAPK p38 in the MCF-7 cell line [140]. The level of SCFAs in the GI tract was measured to be more than 100 mM which is assumed to induce protective changes in the distal organs by the circulatory system [43]. Sodium propionate is an important member in the SCFA family. Park et al., showed that 5 to 20 mM of sodium propionate significantly inhibited proliferation and promoted apoptosis in JIMT-1 and MCF-7 cell lines mediated by inhibited JAK2 STAT3 signaling pathway and cell cycle arrest at G0/G1 phase. Treatment also induced apoptosis by up-regulating the phosphorylated p38 MAKP [38].

SCFAs in extracellular metabolites have been investigated both mixed and individually, according to a study done by Nakkarach et al. [141]. Extracellular metabolites produced by bacterial strain *Escherichia coli* KUB-36 was confirmed to generate seven SCFAs. The MCF-7 BC cell line was treated with SCFA-containing bacterial supernatant and individual SCFAs whose concentration was determined by the actual concentration in the supernatant. Treatment exerted cytotoxicity effects on the MCF-7 cell line but not on the noncancerous control cell line MCF10A. Furthermore, *E. coli* metabolites reduced inflammatory cytokines expression in macrophage cells and promoted anti-inflammatory cytokine IL-10 expression [141]. Although previous studies have investigated the influence of *Lactobacillus* supernatant on BC cells, Nakkarach et al., was the first to show that the mechanism of *Lactobacillus* supernatant depended on anti-inflammation and cytotoxicity effects of SCFAs. Another well-known probiotic strain is *F. prausnitzii*. Similar to *Bifidobacterium longum*, *F. prausnitzii* grows in strict anaerobic conditions and produces high amounts of butyrate and anti-inflammatory compounds. When gut microbiota from BC patients were compared to control, the population ratio of Firmicutes to Bacteroidetes decreased, while the abundance of Verrucomicrobla, Proteobacteria, and Actinobacteria all increased [41]. The abundance of *Faecalibacterium* negatively correlated to phosphorocholine which was up-regulated in cancer patients. Moreover, *F. prausnitzii* can inhibit the IL-6/JAK/STAT3 pathway and inhibit proliferation of the MCF-7 cell line [41]. These results indicate probiotics prevent tumor development by inhibiting cell proliferation and down-regulating IL-6 and JAK whose inhibitors are used for malignancies in clinics [142].

In addition to commercially available probiotics, such as *Lactobacillus* and *Bifidobacterium*, other bacteria species having probiotics effects are also scientifically and clinically important. Hassan et al., explored the anti-cancer impact of heat-killed cells and cytoplasmic fraction of *Enterococcus faecalis* and *Staphylococcus hominis*. The growth of MCF-7 cells was suppressed dose-dependently. Apoptosis and cell cycle arrest at G0/G1 phase was induced after treatment, indicating anti-cancer impact of both bacteria strains in vitro [143].

In addition to using one anti-tumor bacterial metabolite, treating BC cell line MCF-7 with fecal water that contains multiple metabolites was studied by Dubigeon et al. [144]. This experiment closely resembled in situ conditions. Fecal water samples collected from healthy controls or patients newly diagnosed for BC were co-incubated with caco-2 cells to harvest basolateral medium. MCF-7 cells incubated with basolateral medium generated from BC patients had higher viability compared to that from the control. *Bifidobacterium* sp. was positively correlated with cancer cell viability and valerate was the only SCFA that was negatively correlated with viability, suggesting metabolites pooled from BC fecal samples were more likely to increase the risk of BC [144]. Furthermore, lipid metabolism gene expression revealed that *apo AIV* was positively correlated with abundance of Bacteroidetes and SCFA levels, including acetate, propionate, and butyrate [144]. These results open a new direction to study bacterial metabolites in vitro. Fecal water collected from patients need to be metabolized first by gut intestinal epithelial cells, then the basolateral medium was used to treat the BC cell line.

#### 5.1.3. The BC Prevention Roles of Probiotics Metabolites Are Investigated In Vivo

The cancer prevention roles of probiotics are mainly studied in vitro. Exploring the beneficial effects of probiotics in vivo is needed. Cadaverine, a diamines product of lysin decarboxylation, is used by bacteria to buffer environment pH value. Kovacs et al., administered 500 nmol/kg cadaverine to female mice bearing 4T1 BC cells [118]. The treatment successfully reduced tumor growth and primary tumor filtration, as well as attenuated stemness of 4T1 cells. The mechanism of action was mediated by increasing trace amino acid receptors that are known to associate with better survival in BC. Furthermore, cadaverine biosynthesis was reduced in early-stage BC patient fecal samples indicating potential importance of this metabolite in BC prognosis [118]. Park et al., found in vivo sodium propionate at 20 mg/mL significantly prevented tumor development by down-regulating the STAT3 pathway [38]. Studying the mechanistic role of probiotics in animal BC models is needed for developing probiotics supplements and applying probiotics into the translational and clinical fields.

#### 5.1.4. The BC Prevention Roles of Probiotics Are Investigated in Clinical Studies

With emerging studies of probiotics in vitro and in vivo, investigators are exploring the beneficial roles of probiotics in clinical studies. In an observational study, Alcon-Giner et al., orally supplemented preterm infants with *Bifidobacterium* and *Lactobacillus*. Higher fecal acetate, lactate, 2′-fucosyllactose, 3′-fucosyllactose, and arabinose were found in all treatment groups at various time points after birth, and trehalose only in the latest group at 50–99 days after birth. These results showed positive correlation between fecal fatty acids and abundance of probiotic *Bifidobacterium*, the presence of which was further confirmed by 16S rRNA gene profiling and its ability to metabolize oligosaccharides in human milk to acetate [145]. A combination of probiotics and phytochemical was studied by Toi et al., *Lactobacillus casei* strain Shirota and soy isoflavone consumption was evaluated in volunteers aged 40 to 55 in Japan. Though the interaction between probiotics and soy isoflavone was not significant, soy isoflavone consumption was associated with lower BC risk among volunteers [146].

#### 5.1.5. Quorum Sensing between Bacteria Affects BC Progression

Assessing relationships between bacteria is important while modulating gut microbiota for treating BC. Quorum sensing helps bacteria to communicate with other bacteria and respond to environmental stimulation [147]. Bacterial species and host intestinal environment interact to form a dynamic equilibrium. Quorum sensing peptides secreted by gram-positive bacteria are shown to promote angiogenesis and metastasis of BC cells. In addition, quorum-sensing compounds RapG inhibitor produced by *B. subtilis* and competence stimulating peptide, produced by *S. Mitis* found in human intestine, induced angiogenesis and invasion in BC cell lines [148]. On the other hand, quorum-sensing cyclodipeptides produced by some bacteria have antitumor effects [149].

#### 5.1.6. Combinatorial Treatment of Probiotics and Conventional BC Treatment

**Figure 2:**
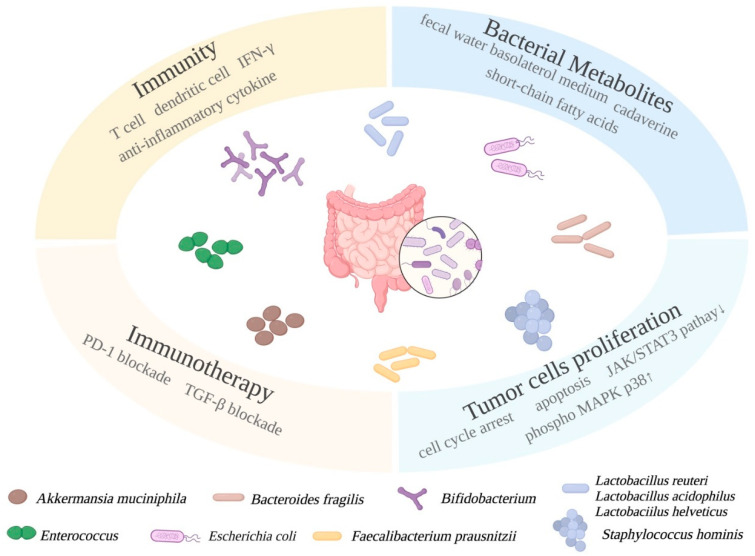
Probiotics and beneficial bacteria with probiotics effects in BC. A higher abundance of ben eficial bacteria was associated with induced protective immune responses and improved conventional immunotherapy treatments. Bacterial metabolite profiles were changed. Anti-cancer mechanistic roles of probiotics were mediated by inhibited tumor cell survival and induced apoptosis.

Recently, investigators have developed combination therapy of probiotics and conventional treatment, such as TGF-beta blockade. Cancer patients developed resistance to a clinical trial drug, galunisertib, a TGF-beta inhibitor [150]. To improve the therapeutic effect of galunisertib, Shi et al., delivered commensal-derived probiotics *E. coli* Nissle 1917 orally to 4T1 and H22 tumors-bearing mice undergoing TGF-beta blockade treatment [151]. Combinatorial treatment successfully inhibited tumor growth and metastasis. Gut microbiota was shaped toward higher abundance of beneficial bacteria, such as genus *Bacteroides*, and genera *Allstipes* and *Allermansia*. Interestingly, *Akkermansia* which was positively correlated with IFN-gamma and negatively correlated to immunosuppressive cytokines [151]. This is direct evidence showing that gut bacteria can enhance immune response. Therefore, the anti-cancer effects of probiotics are mediated by bacterial metabolites and protective immune responses (Figure 2). Probiotics can be used as health supplements to alleviate side effects of chemotherapies and can also be combined to facilitate immunotherapies. 

### 5.2. Prebiotics and Dietary Compounds with Prebiotic Effects in BC Prevention

Prebiotics are dietary mono- and polysaccharides and fibers that promote the proliferation of gut microbiota, especially probiotics. Previous researches have shown that prebiotics can inhibit BC development in various BC cell lines and mouse models. In addition to more commonly seen prebiotics, such as dietary fiber, additional studies recently revealed microbiota changes induced by phytochemicals, such as polyphenols and flavonoids, in diets. Though the prebiotic effect of these phytochemicals needs further exploration, they are shown to promote the growth of microorganisms which are targets of prebiotics. For example, among dietary substrates that have prebiotics effects, polyphenol consumption was associated with a higher abundance of *Bifidobacterium* and *Lactobacillus* and SCFA production in humans [152]. Prebiotics and dietary compounds with prebiotic effects and their mechanisms in BC prevention are discussed in this review (Table 2).

Prebiotics are considered ‘fiber’ and are healthy to consume. Dietary fiber consumption promotes estrogen metabolism and removal of estrogen from the body. Since most BC cases are aggravated by high levels of estrogen, dietary fiber that eliminates hormonal effects has potential for protective effects against BC [43]. Fibers are indigestible in the gut; therefore, gut microbiota can play an important role in digesting fibers and producing metabolites in the GI track environment. Dietary fiber consumption was related to higher prevalence of *Clostridium hathewayi sp*., and insoluble dietary fiber was associated with a higher abundance of *Bacteroides uniformis sp*. in the gut of post-menopausal women newly diagnosed with BC [68]. Despite the beneficial impact of dietary fiber, fermentable oligosaccharides, disaccharides, monosaccharides, and polyols (FODMAP) that are commonly found in foods such as wheat and beans were found to increase overall cancer risk in an epidemiology study focused on breast, prostate, and colorectal cancer [153]. Therefore, consuming prebiotic food with low FODMAP, such as whole grains and tomato, are important for cancer prevention.

#### 5.2.1. The Protective Roles of Mediterranean Diet in BC

Since prebiotics are dietary compounds, food is one of the substantial factors affecting the amounts of prebiotics we consume. Researchers found different dietary patterns shift gut microbiota and can have a positive or negative impact on BC risk. The Mediterranean diet that contains less red meat, refined sugar, carbohydrates, and fat is one of the healthiest dietary patterns. This healthy dietary pattern is associated with fewer cardiovascular disease and cancer risk for all subtypes of BC, while the western diet has the opposite influence. The Mediterranean diet reduces the intake of calories, saturated fatty acid, and amino acids, while it increases the intake of phytochemicals [154]. Therefore, the Mediterranean diet is known to protect against oxidative stress and inflammation and regulate hormones in cancer [154]. It has been hypothesized that gut microbiota also mediates a protective role of this dietary pattern (Table 2). Among people consuming the Mediterranean diet, *Ruminococcus* decreased and *Lachnospira* and *Prevotella* increased in fecal samples [155]. Results were similar in non-human primates in which *Ruminococcus* and *Coprococcus* decreased while *Lactobacillus* and *Clostridium* increased. Shively et al., also showed consuming the Mediterranean diet increased *Lactobacillus* abundance in the mammary gland. The diet reduced oxidative stress metabolites and increased bile acid metabolites which induced an anti-BC effect [119]. Breast milk microbiota can be changed by prebiotics intake. A previous study showed that *S. yanoikuyae* can express glycosphingolipid ligands that activate invariant natural killer T cells [22]. Measuring the microbial sensors TLRs and downstream signaling molecules such as caspase recruitment domain family in breast tissues can also reveal the influence of microbiota on breast tissue [22]. Overall, a healthy diet can effectively increase *Lactobacillus* abundance, bile acid metabolites, and other bioactive compounds in the mammary gland. Phenolic and phytochemical levels also greatly increased after consumption of the Mediterranean diet, though the detailed effects of these chemicals, such as Hippurate, p-cresol sulfate, 3-indole sulfate, and indole propionate, needs further exploration [155].

#### 5.2.2. Phytosterols from Sweet Potato in BC Prevention

Investigators have studied food consumptions ranging from a dietary pattern composed of multiple healthy types of food to one type of vegetable. Though prebiotics generally includes fibers in vegetable and fruit and polysaccharides in wheat bread and vegetables, there are other compounds in food that have been shown to alter gut microbiota population and, potentially, inhibit BC development. Some examples include genistein in soybeans, daucosterol in sweet potato, sulforaphane in broccoli, polyphenols and flavonoids in a variety of vegetable and plants, and piperine in black and white pepper. There is a need to further explore gut microbiota changes in BC models under different dietary compound treatments. Han et al., showed phytosterols extracted from sweet potato, including daucosterol linolenate, daucosterol linoleate, and daucosterol palmitate, inhibited tumor growth and decreased carcinoembryonic antigens in an MCF-7 xerograph mouse model [156]. This prevention effect is associated with increased Bacteroidetes and decreased Firmicutes populations in gut microbiome. The production of SCFAs, such as butyric acid and acetic acid in fecal samples, increased in the treatment groups. Treatment inhibited the PI3K/AKT pathway and NF-kB expression and activated caspases and other pro-apoptotic factors [156]. Overall, the phytosterols from sweet potato shaped gut microbiota and increased SCFA production to lead to inhibited cell cycle progression and apoptosis promotion (Table 2). 

#### 5.2.3. Genistein in BC Prevention

Genistein is an isoflavone in soybeans. Though the effect of genistein has been controversial, dietary supplementation of genistein provides more benefits over negative influence for BC patients under tamoxifen treatment. Paul et al., revealed a significant impact of genistein on the microbiome in a humanized BC mouse model [157]. After treating humanized germ-free mice with 0.25 g/kg of genistein diet, *Lactococcus* and *Eubacterium* abundance increased. After tumor developed, mice on genistein diet had lower abundance of *Ruminococcaceae* [157]. Their result was consistent with other findings where lower abundance of *Ruminococcaceae* was associated with a healthier state (Table 2). Genistein can also prevent BC in offspring born by obese mothers. Dietary genistein after tamoxifen treatment prevented local mammary cancer, reversed negative impact of gut microbiota in offspring, and decreased immunosuppressive mRNA expression in tumors [158].

#### 5.2.4. Grape Polyphenols in BC Prevention

Bioactive compounds, such as polyphenols and flavanols, are well-studied regarding their anti-tumor function. Teixeira et al., delivered polyphenol-enriched grumixama fruit juice to healthy volunteers and obtained a pool of polyphenol metabolites from urine samples. Polyphenol metabolites inhibited cell proliferation and promoted cell cycle arrest at G2/M phase in the MDA-MB-231 cell line [159]. The finding has clinical significance because they used a pool of metabolites from urine samples collected from patients for the cell line experiments. The concentrations and ratios of different polyphenols resemble in situ conditions. Quercetin is an anti-carcinogenic flavanol. C3(1)/SV40 mice fed with 0.2% quercetin in their diet had reduced tumor growth and tumor volume compared to the control [160]. Combined grape polyphenols significantly inhibited BC cell lines proliferation and induced cell cycle arrect in vitro, and greatly reduced primary tumor growth in vivo. Combined grape polyphenols, composed of resveratrol, quercetin, and catechin, had more effective anti-cancer effect in vitro (Table 2). 5 mg/kg of each of the combined compounds significantly reduced tumor growth and metastasis in a MDA-MB-435 xenograft mouse model by up-regulating FOXO1 and NFKBIA [161]. Another well-known commercially available supplementary treatment for BC is piceatannol. Also found in grapes and berries, piceatannol has been shown to regulate transcriptional pathways. Song et al., orally administered 10–20 mg/kg piceatannol in mice carrying 4T1 mammary carcinoma cells [162]. Treatment successfully inhibited tumor growth mediated by down-regulation of STAT3 and p-NFkB p65. Furthermore, treatment induced cell cycle arrest and apoptosis, and led to a decrease in angiogenesis markers, metastasis, and macrophage filtration [162].

#### 5.2.5. Ganoderma Lucidum in BC Prevention

The active component in spores of *Ganoderma lucidum* has been used as a herbal treatment and a health supplement in traditional Asian medicine. Its anti-cancer effect was first evaluated in melanoma and TNBC models. *Ganoderma lucidum* inhibited cell proliferation of MDA-MB-231 cell lines dose-dependently. Moreover, treatment also reduced cell migration and inhibited the release of interleukins and matrix metalloproteinases under pro-inflammatory environment [163]. Another study showed extract from sporoderm-breaking *G. lucidum* (ESG) suppressed 4T1 tumor growth in xenograft mice by stimulating cytotoxic T cell population in both peripheral blood and tumor-infiltrating lymphocytes, as well as down-regulation of inhibitory checkpoints for T cell paradigm. Furthermore, 400 mg/kg ESG-treated mice had altered microbiota composition toward microbiota in normal controls; there was a higher abundance of Firmicutes and Proteobacteria and a lower abundance of Actinobacteria and Bacteroidetes [164]. *Ganoderma lucidum* spore-derived polysaccharide has been combined with the chemotherapy drug paclitaxel in the 4T1 BC mouse model [165]. This combinatorial treatment decreased expression of protein related to the Warburg effect and induced tumor infiltration lymphocytes. More importantly, polysaccharide from *Ganoderma lucidum* reversed gut microbiota dysbiosis caused by paclitaxel treatment; relative abundance of *Bacteroides* and *Ruminococcus* increased and *Ruminococcus* was negatively correlated with fructose-6-phosphate level in tumor [165] (Table 2).

#### 5.2.6. Piperine in BC Prevention

Naturally occurring piperine not only serves as an antioxidant and anti-cancer agent but also shapes gut microbiota. Piperine extract delivered in tablets increased observed species in gut microbiota by 7%; the relative abundance of *Clostridium*, *Bacteroides*, *Enterobacter*, *Enterococcus*, and *Klebsiella* increased compared to the control group, while the relative abundance of *Ruminococcus* and *Blautia* decreased compared to the control group [166]. In addition, piperine is also known to regulate proteins involved in cell cycle and apoptosis pathways, proinflammatory factors and other protein targets in the immune system, in addition to well-studied pathways, such as AKT/mTOR/MMP-9, ERK1/2, and Wnt/Beta-batenin [167]. Based on previous results, regulation of cell proliferation, apoptosis, and immune response were associated with altered gut microbiota (Table 2). However, whether gut microbiome alterations reinforce the anti-cancer function of piperine needs to be further studied.

#### 5.2.7. Bruceae Fructus Oil and Ginko Biloba Leaf in BC Prevention

Bruceae fructus oil (BO) is a vegetable oil derived from *Brucea javanica*. It has been used as a marketable auxiliary treatment for multiple types of cancer, including liver cancer, lung cancer, and gastrointestinal cancer [168,169]. Su et al., first showed the anti-BC effect of BO on TNBC model was dependent on gut microbiome alteration [170]. MDA-MB-231 xenograft mouse model was orally treated 100 to 400 mg/kg BO daily. Several species in the treatment group presented at significantly higher levels, including *C. Melain abacteria*, *N. massiliensis*, and *P. ruminicola*. Changes in microbiota also correlated to changes in metabolic profile. More remarkably, in pseudo germ-free and specific pathogen-free condition (SPF), the tumor-suppression effect of BO decreased. Autophagy was inhibited in non-SPF condition, but not in SPF condition. These findings suggested that the tumor-inhibiting function of BO was dependent on gut microbiota [170] (Table 2). Another group investigated the correlation between gut microbiota and BC resistance proteins (BCRP). Kim et al., found that ginkgo biloba leaf extract suppressed intestinal BCRP expression and modulated gut microbiota composition [171]. The abundance of Bacteroidetes, Firmicutes, and Proteobacteria correlated with intestinal BCRP, among which alpha-Proteobacteria significantly and positively correlated with BCRP expression [171].

#### 5.2.8. Marine Originated Compounds in BC Prevention

Polymers derived from plants can promote the growth of probiotics. Therefore, they are proposed as potential prebiotics used in the food industry and as health supplements. These prebiotics candidates can be used for developing pharmaceutical treatments or supplements in the future. A marine-origin compound, fucoidan, has a sulfated poly-alpha-(1-3)-fucopyranoside long backbone with branched single units. Fucoidan is enriched in brown seaweed which is consumed frequently in East Asian diets. Xue et al., found that in 7,12-dimenthybenz anthracene induced BC rat model and that 200 mg and 400 mg per kg body weight fucoidan recovered damaged intestinal wall and increased diversity of intestinal microbiota with increased Bacteroidetes-to-Firmicutes ratio [172]. Furthermore, plasma endotoxin, D-lactic acid, and diamine oxidase decreased in treatment groups. The expression of phosphorylated p38 MAPK and ERK1/2, occludin, claudin-1, and claudin-8 were up-regulated [172]. These results associated beneficial gut microbiota alteration with pro-apoptotic function of fucoidan and better overall survival outcome, as a low level of claudin is a critical marker for primitive breast malignancies (Table 2). Another dietary compound found in marine fish oil that has prebiotics effect is n-3 polyunsaturated fatty acids (PUFAs) [173]. Well-known anti-cancer and antioxidant omega-3 and omega-6 are major classes of PUFAs. Vijay et al., compared the prebiotic effects of omega-3 with inulin; inulin increased the abundance of *Bifidobacterium* and *Lachnospiraceae*, while omega-3 increased the abundance of *Coprococcus spp*. and *Bacteroides spp*. These two compounds also changed metabolic profiles differently [174]. Moreover, maternal PUFAs consumption was shown to decrease tumor incidence and shape gut microbiota in offspring [175]. Maternal generation was fed fish oil-supplemented diet during gestation and lactation, and all offspring were on normal diet. Gut microbiota of offspring in the maternal-treated group had higher alpha diversity; the abundance of *Akkermansia*, *lactobacillus*, and *Mucispirillum* increased. Interestingly, the abundance of *Mucispirillum* was positively associated with anti-inflammatory factor, while the abundance of *Akkermansia* was negatively correlated with pro-inflammatory factors [175] (Table 2). These previous results highlighted the importance of correlation studies that associate gut microbiota alteration with clinically significant factors such as inflammation and gastrointestinal dysbiosis as well as age, sex, and other factors.

#### 5.2.9. Ginseng in BC Prevention

Ginseng, a traditional herbal medicine frequently used in East Asian countries, has anti-tumor effects against BC and lung cancer models and is known to have therapeutic effects on diabetes, colitis, and obesity [176,177]. Ginseng polysaccharides treatment promoted the growth of probiotics *Bifidobacterium*, *Bacteroides*, and *Verrucomicrobia*, while it reduced pathogenic bacteria in gut microbiota [178]. Huang et al., found ginseng altered gut microbiota and increased metabolite valeric acid while it decreased kynurenine/tryptophan ratio to activate regulatory T cells which sensitize non-small cell lung cancer to anti-PD-1 therapy [179]. BC tumor microenvironment can also be potentially remodeled by ginseng polysaccharides (Gp). For example, Gp was shown to enhance cytotoxicity of natural killer cells and stimulate dendritic cells in murine bone marrow [180,181]. The gut environment associated with mammary carcinogenesis was improved by ginsenoside. The expression of tight junction proteins was up-regulated to protect the barrier of the small intestine. In addition to Gp, ginseng contains a variety of compounds, such as Rg3 and Rd, for inhibiting angiogenesis and suppressing cancer stemness, and Rh2 that modulate immune response [182]. Though the role of ginseng and its compounds in gut microbiota alteration and BC prevention remains unclear, ginseng polysaccharide and other ginsenosides may be promising dietary supplements or treatment agents (Table 2).

#### 5.2.10. Poria Cocos in BC Prevention

Poria cocos (PC) is a traditional East Asian health product and medicine frequently consumed in the diet and added as a supplementary product in yogurt and staple food. PC belongs to the Polyporaceae fungus family and has been used as an effective treatment for gastrointestinal disorders. Jiang et al., applied 700 mg/kg PC to xenograft mice carrying MDA-MB-231 cancer cell tumors [183]. Treatment greatly reduced tumor growth and repaired intestinal barrier dysfunction. PC protectively shaped gut microbiota composition; *Lactobacillus* and *Bifidobacterium*, known as probiotics, both increased, while *Mucispirillum* and *S24-7* were related to reduced inflammation status [183]. Moreover, *Bacteroidetes* positively correlated with tight junction proteins and markers of intestinal barrier, indicating poria cocos had a protective impact on GI health in TNBC (Table 2).

#### 5.2.11. Prebiotic Inulin in BC Prevention

Another well-studied prebiotic is inulin, a non-digestible polysaccharide that can be utilized by gut microbiota, including probiotics such as *Bifidobacterium*, to produce SCFAs [184]. Dietary inulin improved glucose metabolism in gestational diabetes by up-regulating the insulin signaling pathway [185]. Inulin was also shown to decrease caloric intake and improve glucose tolerance in a dose-dependent manner [186]. The microbiota and metabolites were both altered; the relative abundance of Bacteroidetes and *Bifidobacterium spp*. both increased and protective metabolites, such as butyryl-CoA and peptide YY, increased in the cecum [186]. Regarding the anti-cancer efficacy of inulin, Taper et al., showed 15% (w/w%) inulin or oligofructose reduced tumor growth and metastasis in a mammary mouse carcinoma EMT6 model [187]. In an ER+ BC mouse model, MCF-7 athymic xenograft was stimulated by 17 beta-estradiol. 5% (w/w%) fructooligosaccharides supplemented in food inhibited tumor growth and induced apoptosis mediated by decreased expression of bcl-2, bcl-xL, and cyclin D1 [188]. These results suggested inulin alone can attenuate tumor development. The anti-cancer effect of inulin is strengthened when combined with probiotics *Lactobacillus plantarum* [189,190]. Combinatorial treatment induced protective immune response, increased CD4^+^ T-cells in tumor and decreased tumor necrosis factor-alpha in serum [189]. 20 g/kg probiotic strain *L. plantarum* LS/07 and 20 mg/L oligofructose-enriched inulin were delivered to rats bearing BC tumor through daily diet and water, respectively. The ratio of high-grade to low-grade carcinomas and Ki-67 expression decreased in combinatorial treatment [190] (Table 2).

#### 5.2.12. Combinatorial Dietary Treatments in BC Prevention

As we have studied the influence of single dietary compounds on BC models, investigators have recently begun to observe the combinatory effect of two or more compounds. In combinatorial treatment, fewer dosages of single compounds are combined and elicit greater effect on gut microbiota modulation and cancer prevention. Sharma et al., studied the combinatorial impact of green tea polyphenols and broccoli sprouts on gut microbiota and short-chain fatty acids levels in a Her2/neu transgenic mouse model (Table 2). The relative abundance of *Adlercreutzia*, *Lactobacillus*, and *Lachnospiraceae*, and *S24-7* increased in treatment groups. Interestingly, the concentration of plasma SCFAs, including propionate and isobutyrate, increased in treated mice [191]. In another study, a combination of polyphenols, flavonoids, and functional proteins in natural nanovehicles from tea flowers (TFENs) had great anti-tumor effect both in vitro and in vivo [192] (Table 2). These nanovehicles have a lipid layers exosomal-like structure and a large amount of polyphenols and flavonoids inside lipid layers. The cytotoxicity effect against BC cell lines of TFENs was mediated through stimulated reactive oxygen species (ROS). ROS resulted in increased cleaved caspases, decreased cyclin A and B in cell cycle arrest, and anti-migration and pro-apoptotic activities in MCF-7 and 4T1 BC cell lines. The cancer-prevention effect of TFENs was also shown in vivo in a MCF-7 xenograft mouse model. Oral treatment or intravenous injection significantly decreased tumor weight and volume, and inhibited metastasis. More importantly, gut microbiota was greatly altered after treatment. The relative abundance of *Muribaculaceae*, *Lachnospiraceae NK4A136*, *Dubosiella*, and *Desulfovibrionaceae* increased, while the relative abundance of *Bacteroides*, *Prevotellaceae*, and *Roseburia* decreased [192].

**Table 2:** Prebiotics and dietary compounds with prebiotic effects induce gut microbiota alterations which are associated with anti-cancer mechanisms in BC.

Dietary Compounds and Prebiotics	Study Design	Sequencing Method	Gut Microbiota Alteration	Associated Anti-Cancer Mechanism	References
Genistein	Germ-free RAG2−/− humanized mice were treated with 0.25 g/kg genistein-supplemented diet; mice with ER+ mammary tumors were treated with 500 ppm genistein diet with or before tamoxifen treatment.	High throughput 16S rRNA	Phylum *Verrucomicrobia* significantly changed. Family *Lachnospiraceae* and *Ruminococcaceae* increased. The abundance of genera *Lactococcus* and *Eubacterium* increased; Genistein decreased *Prevotellaceae* and *Enterobacteriaceae*, increased SCFA producing *Clostridiaceae*.	Genistein metabolism changed in mice humanized with post-chemotherapy patient fecal samples. 4-ethylphenol and 2-(4-hydroxyphenol) propionic acid were completely depleted. BC tumor latency was 25%. Tumor size significantly decreased; Genistein reduced genotoxic tyramine levels, increased tumor suppressor gene expression, and induced protective immunity changes.	[157,158]
Piperine	Healthy volunteers consumed turmeric tablets and curcumin tablets, both contained 1.25 mg black pepper BioPerine. Piperine was used to treat BC stem cells and Her2-over-expressed cells.	High throughput 16S rRNA	The abundance of *Clostridium* spp., *Bacteroides* spp., *Citrobacter* spp., *Cronobacter* spp., etc., increased; the abundance of *Blautia* spp. and *Ruminococcus* spp. decreased.	Piperine decreased mammosphere formation, decreased aldehyde dehydrogenase expression, and inhibited Wnt related pathway in vitro. Piperine also inhibited cell proliferation and induced apoptosis by inducing caspase-3 and PARP cleavage.	[166,167]
Fucoidan	Rats with DMBA-induced tumors were orally treated with 200 or 400 mg per kg body weight fucoidan.	High throughput 16S rRNA	Intestinal microbiota diversity increased. Bacteroidetes/Firmicutes phylum ratio increased. The abundance of *Prevotella*, *Blautia*, and *Parabacteroides* increased. The abundance of *Coprococcus*, *Oscillospira*, *Collinsella*, etc., decreased.	In plasma metabolite profile, D-lactic acid and diamine oxidase decreased. Expression of occludin and claudins increased. Expression of phosphor-MAPK p38 and ERK1/2 increased.	[172]
n-3 polyunsaturated fatty acids	C57BL/6 pregnant mice were treated with fish oil supplemented diet or flaxseed oil supplement diet or n-3 PUFA deficient diet. Offspring were on normal diet.	High throughput 16S rRNA	n-3 PUFA diet increased microbial diversity. The abundance of *Akkermansia*, *Lactobacillus*, and *Mucispirillum* increased. In n-3 PUFA deficient group, the abundance of *Lactobacillus*, *Bifidobacterium*, and *Barnesiella* decreased.	n-3 PUFA treatment reduced pro-inflammatory IL-1β, IL-6 and TNF-α. The abundance of *Bifidobacterium* was negatively associated to IL-1β and IL-6. The abundance of *Mucispirillum* was positively associated with IL-10. n-3 PUFA deficient group had lower level of butyric acid metabolism.	[175]
Inulin	Rats were orally treated with different concentrations of inulin, ranging from 2.5% to 25%. Rats with DMBA-induced tumors were treated with 20 g/kg prebiotics oligofructose-enriched inulin, or in combination with probiotics and/or melatonin.	High throughput 16S rRNA	Increase in abundance of *Bifidobacterium* and *Lactobacillus* in multiple studies. The abundance of Bacteroidetes and *Bifidobacterium* spp. increased. *Clostridium* decreased.	Inulin decreased caloric intake dose-dependently. In plasma metabolite profile, butyryl-CoA to acetate CoA-transferase increased, and plasma peptide YY increased. The expression of Ki-67 marker decreased. CD4^+^ and CD8^+^ T cells and regulatory T cells were induced.	[184,185,186,187,188,189,190]
Sweet potato (daucosterol, linolenate, and daucosterol)	BALB/C MCF-7 xenograft mice were treated with 87.8 mg/kg/day sweet potato extracted mixed compounds.	High throughput 16S rRNA	The abundance of Bacteroidetes increased, Firmicutes decreased. At family level, S24-7 increased while *Ruminococcaceae* decreased. At genus level, the abundance of *Lachnospiraceae*, *Alistipes* and *Ruminiclostridium_5* decreased, according to different compounds.	Gut microbiota was associated with anti-tumor effect. In tumor tissue, caspase 3 and PARP1 cleavage was induced, PI3K/AKT/NF-κB pathway was inhibited. Ki67 and VEGF were down-regulated. Apoptosis was induced.	[156]
*Ganoderma* *lucidum* (SGP)	4T1 BC mouse model was orally treated with 200 or 400 mg/kg SGP. Combinatorial treatment was SGP with paclitaxel.	16S rRNA	SGP restored gut microbiota dysbiosis induced by paclitaxel. The abundance of *Bacteroides*, *Ruminococcus*, etc., increased, while *Desulfovibrio* and *Odoribacter* decreased.	Correlation analysis revealed the abundance of *Ruminococcus* was negatively correlated to fructose-6-phosphate level in tumor tissue. Treatment down-regulated Warburg effect-related targets. Treatment also induced tumor infiltration lymphocytes and other protective immune responses, such as inhibited IL-8, IL-6, MMP2, and MMP9.	[163,164,165]
Bruceae fructus oil (BO)	BALB/c MDA-MB-231 xenograft mice were orally treated with 100, 200, or 400 mg/kg BO for 30 days. For germ-free experiment, mice were treated with 400 mg/kg BO for 20 days.	Metagenomics (And untargeted metabolomics)	Microbiota diversity was not significantly affected. The abundance of *Candidatus Melainabacteria bacterium MEL.A1*, *N. massiliensis*, and *P. ruminicola* greatly increased compared to the control group.	BO had no cytotoxicity effect on MDA-MB-231 cell line. Tumor suppression effect depended on microbiota alteration because anti-tumor effect was not seen in germ-free condition. Untargeted metabolomics of amino acid profile in serum showed L-(-methionine) and L-threonine decreased under BO treatment. They negatively correlated to species enriched by BO treatment. The abundance of *Candidatus Melainabacteria bacterium MEL.A1* negatively correlated to several targets in amino acid metabolism. Tumor mTOR activity was up-regulated. Autophagy process was restrained.	[170]
Poria cocos (PC)	BALB/c MDA-MB-231 xenograft mice were gavaged with 100 μL/10 g weight PC.	16S rRNA	The abundance of *Lactobacillus* and *Bifidobacterium* increased, while *Desulfovibrio*, *Mucispirillum*, *S24-7*, and *Straphylococcus* decreased.	PC enhanced tight junction proteins and up-regulated ERK1/2 and p38 MAPK levels to strengthen intestinal barrier function in BC model. *Prevotella*, *Rikenellaceae*, and Bacteroidetes were correlated to p38 MAPK and claudin expression.	[183]
Ginseng polysaccharides (GPs)	C57BL/6 mice carrying Lewis lung cancer cells were treated with GPs combined with αPD-1 monoclonal antibody. Fecal pellets of non- responders were transferred to germ-free mice. Germ-free mice were treated with combined GPs and αPD-1 antibody.	16S rRNA (And UPLC-MS)	GPs group had increased abundance of *Muribaculaceae*. αPD-1 responders and non-responders had different gut microbiome. GPs rescued non-responders and increased the abundance of *Parabacteroides disitasonis* and *Bacteroides vulgatus*; Ginseng treatment can increase the abundance of probiotics, such as *Bifidobacterium* and *Akkermansia*, while reducing pathogenic bacteria, such as *Deferribacters* and *Helicobacter*.	GPs treatment increased serum valeric acid level and decreased serum L-kynurenine level. Regulatory T cells and effector T cells were suppressed, thereby sensitizing mice to αPD-1 blockade treatment. Ginsenoside and cyclophosphamide inhibited NF-κB pathways and up-regulated caspase-3 and Nrf2. The expression of tight junction proteins was promoted to protect intestinal barrier in mammary carcinoma. Tumor suppressors were up-regulated, and protective immune responses were induced by ginseng-derived compounds.	[179,180,181]
Combinatorial green tea polyphenols (GTP) and broccoli sprout (BSp)	Her2/neu mouse model was orally fed 26% (*w*/*w*) BSp, 0.5% (*w*/*v*) GTP, and in combination. Treatment started from maternal gestation stage and continued in offspring.	16S rRNA (And LC-MS)	The abundance of *Adlercreutzia*, *Lactobacillus*, such as *L. reuteri*, and *Lachnospiraceae* increased. Combinatorial treatment increased the abundance of *Akkermansia* *muciniphila* and decreased the abundance of *Lactococcus*.	Cross-generation BSp and GTP treatment altered gut microbiota. SCFA levels in plasma were measured by LC-MS. Combinatorial treatment increased propionate and isobutyrate levels in plasma compared to control group.	[191]
Tea flowers nanovehicles (TFENs)	BALB/c MCF-7 xenograft mice were treated with 1.5 or 3 mg protein/kg TFENs by intravenous injection (i.v.) or oral delivery.	16S rRNA	Orally treated group had higher Firmicutes to Bacteroidetes ratio. The abundance of *Blautia* and *Alistipes* were higher in i.v. and oral treated groups compared to control.	TFENs promoted reactive oxygen species levels to inhibit cell proliferation in vitro. TFENs inhibited BC tumor grow and metastasis. Tumor volume and tumor weight were significantly reduced under treatment. Lung metastasis and the number of metastasis nodules greatly decreased under treatment.	[192]

#### 5.2.13. Secondary Bile Acid in BC Prevention

In addition to prebiotics and probiotics, bile acids also have an important role in BC prevention. Lithocholic acid (LCA) is a bile acid that solubilizes fats for absorption. Chenodeoxycholic acid is reduced to form LCA by bacteria in the colon. Since mammary tumor cells have increased lipogenesis, Luu et al., evaluated the inhibitory effect of LCA on BC cells [193]. 50 uM to 200 uM of LCA induced TGR5 expression and pro-apoptotic factors in both MCF-7 and MDA-MBA-231 cells. LCA increased p53 expression and decreased Bcl-2 expression in MCF-7 cells. More importantly, regarding the inhibitory effect against lipogenesis, LCA decreased expression of SREBP-1c, FASN, and ACACA [193]. Another study focused on LCA also showed its ability to reduce proliferation by inhibiting Warburg metabolism and decreasing aggressiveness by inhibiting endothelial-to-mesenchymal transition [120]. These studies open a new direction for cancer prevention investigations. Though bile acid has been studied for its importance in lipid and glucose metabolism, vitamin absorption, and energy homeostasis, its role in cancer prevention needs to be further explored in cell lines as well as animal models for developing potential treatment methods.

**Figure 3:**
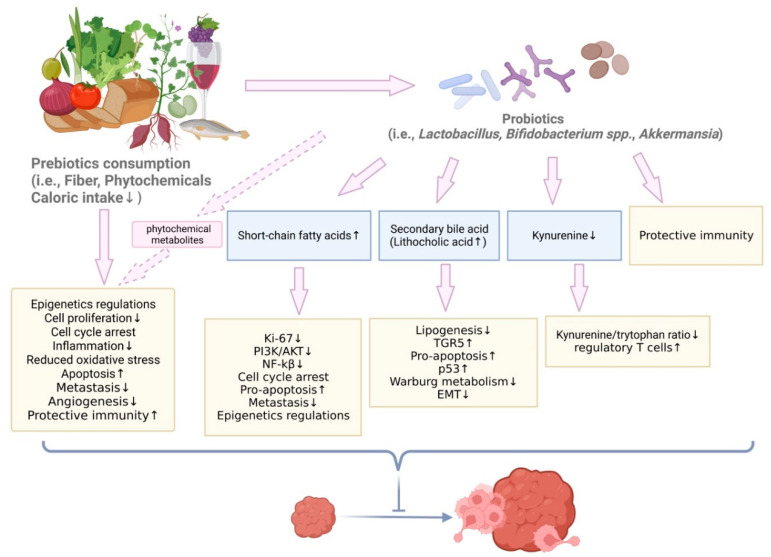
Prebiotics and dietary supplements having prebiotic effects in BC. Prebiotics consumption increased fiber and bioactive phytochemicals levels in situ and promoted the growth of probiotics. Tumor size, weight, metastasis, and EMT were reduced by protective bacterial metabolites, reduced pro-inflammatory status, up-regulated tumor-suppressor genes, and inhibited tumor cell proliferation.

Therefore, prebiotics and a variety of dietary compounds that have prebiotic effects have been studied regarding their anti-BC mechanisms which are mediated by bacterial metabolites, bile acids, and protective immune responses. Gut microbiota population can be altered by prebiotics and dietary compounds. Microbiota changes have been associated with biological processes (Table 2). Biological processes, such as reduced tumor cells proliferation, inhibited oxidative stress, induced apoptosis, and decreased metastasis, all contribute to slowing down BC tumor development and cancer prevention (Figure 3).

## 6. Epigenetics Impact of Microbiota Altered by Nutrients

Microbiota can have protective or destructive roles in BC development mediated by epigenetics regulations. Besides many other factors, such as genotoxins, inflammation, and immunity, epigenetics is another potential mechanism connecting microbiota and BC [13,14]. Previous research showed that microbiota can influence DNA methylation and regulate DNA damage and repair [91]. Epigenetics modulation leads to up- or down-regulated cellular growth or signaling pathways. Therefore, studying epigenetics as one mechanism for microbiota to affect BC is very important. *Fusobacterium nucleatum* express FadA protein to regulate E-cadherin and Beta-catenin pathways to promote colon cancer [194]. *E. coli* is known to induce inflammation DNA damage, such as DNA double-strand breaks in colorectal cancer [195,196]. However, epigenetics regulation by microbiota in BC remains unclear. Epigenetics mechanisms include DNA methylation, histone proteins acetylation and deacetylation, and modification on non-coding RNAs, such as long noncoding RNA and miRNAs. Diet and phytochemicals contained in food can regulate epigenetics enzymes and related pathways [197]. The expression of genes instead of genome are altered and can be inherited in the next generation [198]. During DNA methylation, a methyl group is transferred from donor to cytosine bases in CpG islands [199]. Hypermethylation in BC has been related to increased risk of BC due to inhibition of tumor-suppressor genes. Histone proteins can also be methylated, acetylated, phosphorylated, and ubiquitinated, among which histone acetylation has gained more attention in the field of microbiology in BC. Butyrate can be produced by a variety of bacteria, such as *F. prausnitzii* and *Clostridium* spp. The epigenetics regulatory role of microbiota is mediated by immunity and secreted metabolites. Secreted compounds and metabolites can alter enzymes involved in methylation or acetylation processes. In addition to FDA-approved HDAC inhibitors, bacterial metabolites, such as SCFAs, isothiocyanates, folate, and biotin, have also been studied as epigenetics regulators. Isothiocyanates can be generated from glucosinolates by gut bacteria, such as *Enterococcus faecalis*, *Bacteroides thetaiotaomicron*, and *Peptostreptococcus sp.* [200]. Many SCFAs are known as HDAC inhibitors. Butyrate that serves as an HDAC inhibitor induced cancer cell death, promoted apoptosis, and inhibited migration in BC cell lines [201,202]. It can also induce epigenetic changes to activate tumor-suppressor genes such as *p21*. Dietary polyphenol epigallocatechin-3-gallate (EGCG) and isothiocyanate sulforaphane (SFN) can change gut microbiota composition and regulate epigenetics markers [203]. EGCG is a polyphenol extracted from green tea with health beneficials. EGCG inhibits DNA methyltransferase (DNMT) in BC prevention [204]. Metabolized by gut microbiota from glucoraphanin, SFN is an anti-oxidant, anti-tumor agent, HDAC and DNMT inhibitor, and hTERT inhibitor [205,206,207]. Sharma et al., showed combinatorial dietary compounds SFN and EGCG changed gut microbiota and elevated SCFAs levels in a Her2/neu transgenic mouse model [191]. The same combinatorial treatment induced cell cycle arrest and epigenetics modulation through decreased expression of DNMT1, DNMT3a, DNMT3b, and HDACs in vivo [208]. Therefore, changes in bacteria composition and metabolic profile have been associated with epigenetics regulations.

As an important component of epigenetics regulation, non-protein coding miRNAs have been studied for their interaction with gut microbiota. miRNAs can regulate gene expression by degrading multiple mRNAs and interfering with translation that regulates tumor cell proliferation and survival, metastasis, and other important cancer-related biological processes in BC [209,210]. Recently, investigators have found association between microbiota alteration and miRNAs expression in various types of cancer [211]. For example, miR-515-5p and miR-1226-5p can not only target the nucleic acid sequences but also promote the growth of *Fusobacterium nucleatum* and *E. coli*, respectively [212]. Bidirectional interaction between gut microbiota and host miRNA expression was established where gut microbiota affects miRNA expression through MyD88-dependent pathway and TLR4 signalings while host miRNAs can be taken up by gut microbiota and affect the growth of bacteria [209]. Several miRNAs’ expressions are distinct in healthy individuals and BC patients. miR-21 is over-expressed in breast tumor samples while other miRNAs, such as miR-126 and miR-199a, were under-expressed [213]. Different miRNAs were also identified in different subtypes of BC [209]. Circulating miR-21 has gained more attention in the field because it is used as a biomarker to recognize metastasis levels, diagnosis, and prognosis. miR-155 was studied as a prognosis biomarker in early-stage TNBC [209]. The mechanistic role of miRNAs depends in part on host immune regulations as some miRNAs can help tumor cells to escape host immune surveillance. In the microenvironment of gut and breast tumors, inflammation status, miRNA expressions, and microbiota population are closely regulated mutually. Since miRNAs can regulate innate and adaptive immunity, their expression may affect the outcome of immune checkpoint blockade therapies [214]. Gut microbiota also influences the effectiveness of PD-1 blockade. For example, higher abundance of *Enterococcus*, *Ruminococcaceae*, and *Akkermansia* were found to increase the effectiveness of PD-1 blockade indicating that miRNA and gut microbiota are acting simultaneously in immune-based therapies for BC [136]. Studying mechanistic roles of miRNA and gut microbiota helps investigators to develop miRNA-based therapies for BC.

Epigenetics regulations of gut and breast microbiota in BC prevention, treatment, and reoccurrence need further exploration. This plays an important role in cancer prevention because early-life dietary treatment showed promising anti-cancer effects of phytochemicals [207]. Despite research in microbiota and epigenetics in separate fields making connections between gene expression and the environment, epigenetics and diets in carcinogenesis will help increase understanding of the mechanistic roles of microbiota, especially when applied to facilitate miRNA-based therapies and conventional BC treatment

## 7. Conclusions

Gut microbiota and breast microbiota play critical roles in healthy individuals and BC patients. Dysbiosis of microbiota in breast tissue and the GI track are both associated with a higher risk of BC. Although the association between microbiome and development of BC has been studied in the past decade, there are many remaining questions about the mechanistic role of specific bacterial strains in carcinogenesis progression. Serving as supplements for conventional treatments, probiotics strains and dosage and the mechanism for facilitating conventional treatments have not been studied in depth. Further studies are needed to reveal cause and effect mechanisms between microbiota and BC. Although modulating microbiota with probiotics and/or prebiotics are promising strategies of cancer prevention, there are many open avenues for invention, such as resolving the interactions between particular strains and the host, whether microbiota alteration leads to BC or vice versa, whether dysbiosis can be reversed by probiotics or prebiotics in patients, and the dosage and timing of supplementary treatment. Conducting experiments involving various animal models and human volunteers is encouraged in this field. Precision medicine is also critical when addressing these remaining questions as each individual has distinct microbiota composition. More large-scale and proof-of-concept research need to be done before research findings can be brought to the translational and clinical stages.

## Figures and Tables

**Table 1:** Gut microbiota and breast microbiota produce fatty acid metabolites that have anti-cancer effects.

Bacteria	Location	Fatty Acid Metabolite	Associated Anti-Cancer Mechanism	References
*Bifidobacterium* spp. (Actinobacteria)	gut	Docosahexaenoic acid, Eicosapentanoic acid, Omega-3 PUFA, acetate, and formate	Reduces expression of oncogenic miRNA-21, increases PTEN expression, suppresses cell proliferation by modulating Bcl-2 and procaspase-8.	[30,31,32]
*Lactobacillus* spp. (*Lactobacillaceae*)	gut, breast	Conjugated linoleic acid, butyrate, acetate, and lactate	Activates tumor suppressor genes *p53*, *p21* and *p27*, suppress VEGF, induces G0/G1 cell cycle arrest and apoptosis.	[33,34,35,36]
*Akkermansia municiphila* (Verrucomicrobia)	gut	Propionate, and acetate	Inhibits JAK/STAT3 pathway, activates p38 through oxidative phosphorylation and, therefore, promotes G0/G1 cell cycle arrest.	[37,38]
*Bacillus cereus* (Firmicutes)	gut	5-α -pregnane-3,20-dione, and butyrate	Derived from progesterone metabolism, metabolite promotes cancer progression via increase in cell proliferation.	[39]
*Bacillus subtilis* and *Bacillus lichenofirmes* (Bacillota)	gut	Propionate	Down-regulate STAT3 pathway.	[40]
*Faecalibacterium praunitzii* (*Ruminococcaceae*)	gut, breast	Butyrate	Suppress cancer by inhibiting IL-6 and phosphorylation of JAK/STAT pathway proteins by promoting apoptosis and decreasing cell proliferation.	[41]
*Blautia* spp. (*Lachnospiraceae*)	gut	Acetate, formate, and propionate	NA	[32]
*Propionibacterium* (Actinomycetota)	breast	Acetate, and propionate	NA	[42]
*Clostridium* (Firmicutes)	gut	Acetate, butyrate, propionate, valerate, formate, and lactate	NA	[32]
*Streptococcus* (Bacillota)	breast	Acetate, formate, and lactate	NA	[32]
*Coprococcus* and *Roseburia* (Lachnospiraceae)	gut	Butyrate	Induce G2/M cell cycle arrest. Inhibit HDAC1 and HDAC3, activate GPR109A and GPR43, thereby reducing inflammation and suppressing tumor by over-expressing p57, reduces neuropilin-1 and ERK-MAPK pathway protein expression that prevents angiogenesis, metastasis and proliferation, triggers apoptosis via Wnt/β-catenin signaling and suppresses c-Myc.	[43]
*Prevotella* (Bacteroidota)	gut	Acetate, formate, and propionate	NA	[32]
*Bacteroides* spp. (Bacteroidota)	gut	Valerate	Modulate DNMT activity thereby hypermethylating *HDAC6*, *NASP*, *HNRNPC*, and *LIN9* genes altering their expression which, in turn, promotes hallmarks of breast cancer progression.	[44,45]

Abbreviations: PUFA, polyunsaturated fats; PTEN, Phosphatase and tensin homolog; VEGF, vascular endothelial growth factor; JAK-STAT, Janus kinase-signal transducer and activator of transcription; IL, interleukin; HDAC, histone deacetylase; GPR, G-protein coupled receptors; ERK, extracellular signal-regulated kinases; MAPK, mitogen-activated protein kinase; c-Myc, cellular myelocytomatosis oncogene; NASP, nuclear autoantigenic sperm protein; HNRNPC, heterogeneous nuclear ribonucleoproteins C1/C2; and LIN9, Lin-9 homolog.

## Data Availability

Not applicable.

## References

[1] Plottel C.S., Blaser M.J. (2011). Microbiome and malignancy. Cell Host Microbe.

[2] Sommer F., Anderson J.M., Bharti R., Raes J., Rosenstiel P. (2017). The resilience of the intestinal microbiota influences health and disease. Nat. Rev. Microbiol..

[3] Ruo S.W., Alkayyali T., Win M., Tara A., Joseph C., Kannan A., Srivastava K., Ochuba O., Sandhu J.K., Went T.R. (2021). Role of gut microbiota dysbiosis in breast cancer and novel approaches in prevention, diagnosis, and treatment. Cureus.

[4] Silva Y.P., Bernardi A., Frozza R.L. (2020). The role of short-chain fatty acids from gut microbiota in gut-brain communication. Front. Endocrinol..

[5] Yao Y., Cai X., Fei W., Ye Y., Zhao M., Zheng C. (2022). The role of short-chain fatty acids in immunity, inflammation and metabolism. Crit. Rev. Food Sci. Nutr..

[6] Thomas S.P., Denu J.M. (2021). Short-chain fatty acids activate acetyltransferase p300. eLife.

[7] Yang H., Pinello C.E., Luo J., Li D., Wang Y., Zhao L.Y., Jahn S.C., Saldanha S.A., Planck J., Geary K.R. (2013). Small-molecule inhibitors of acetyltransferase p300 identified by high-throughput screening are potent anticancer agents. Mol. Cancer Ther..

[8] Rea D., Coppola G., Palma G., Barbieri A., Luciano A., Del Prete P., Rossetti S., Berretta M., Facchini G., Perdonà S. (2018). Microbiota effects on cancer: From risks to therapies. Oncotarget.

[9] O’Hara A.M., Shanahan F. (2006). The gut flora as a forgotten organ. EMBO Rep..

[10] Sender R., Fuchs S., Milo R. (2016). Are we really vastly outnumbered? revisiting the ratio of bacterial to host cells in humans. Cell.

[11] Luu T.H., Michel C., Bard J.M., Dravet F., Nazih H., Bobin-Dubigeon C. (2017). Intestinal proportion of Blautia sp. is associated with clinical stage and histoprognostic grade in patients with early-stage breast cancer. Nutr. Cancer.

[12] Terrisse S., Derosa L., Iebba V., Ghiringhelli F., Vaz-Luis I., Kroemer G., Fidelle M., Christodoulidis S., Segata N., Thomas A.M. (2021). Intestinal microbiota influences clinical outcome and side effects of early breast cancer treatment. Cell Death Differ..

[13] Garrett W.S. (2015). Cancer and the microbiota. Science.

[14] Schwabe R.F., Jobin C. (2013). The microbiome and cancer. Nat. Rev. Cancer.

[15] Velicer C.M., Heckbert S.R., Lampe J.W., Potter J.D., Robertson C.A., Taplin S.H. (2004). Antibiotic use in relation to the risk of breast cancer. JAMA.

[16] Mayo B.J., Secombe K.R., Wignall A.D., Bateman E., Thorpe D., Pietra C., Keefe D.M., Bowen J.M. (2020). The GLP-2 analogue elsiglutide reduces diarrhoea caused by the tyrosine kinase inhibitor lapatinib in rats. Cancer Chemother. Pharmacol..

[17] Roy S., Trinchieri G. (2017). Microbiota: A key orchestrator of cancer therapy. Nat. Rev. Cancer.

[18] Yang P., Wang Z., Peng Q., Lian W., Chen D. (2021). Comparison of the gut microbiota in patients with benign and malignant breast tumors: A pilot study. Evol. Bioinform. Online.

[19] Bobin-Dubigeon C., Luu H., Leuillet S., Lavergne S., Carton T., Le Vacon F., Michel C., Nazih H., Bard J.-M. (2021). Faecal microbiota composition varies between patients with breast cancer and healthy women: A comparative case-control study. Nutrients.

[20] Okubo R., Kinoshita T., Katsumata N., Uezono Y., Xiao J., Matsuoka Y.J. (2020). Impact of chemotherapy on the association between fear of cancer recurrence and the gut microbiota in breast cancer survivors. Brain Behav. Immun..

[21] Guan X., Ma F., Sun X., Li C., Li L., Liang F., Li S., Yi Z., Liu B., Xu B. (2020). Gut microbiota profiling in patients with HER2-negative metastatic breast cancer receiving metronomic chemotherapy of capecitabine compared to those under conventional dosage. Front. Oncol..

[22] Xuan C., Shamonki J.M., Chung A., DiNome M., Chung M., Sieling P.A., Lee D.J. (2014). Microbial dysbiosis is associated with human breast cancer. PLoS ONE.

[23] Chan A.A., Bashir M., Rivas M.N., Duvall K., Sieling P.A., Pieber T.R., Vaishampayan P.A., Love S.M., Lee D.J. (2016). Characterization of the microbiome of nipple aspirate fluid of breast cancer survivors. Sci. Rep..

[24] Buchta Rosean C., Bostic R.R., Ferey J.C.M., Feng T.Y., Azar F.N., Tung K.S., Dozmorov M.G., Smirnova E., Bos P.D., Rutkowski M.R. (2019). Preexisting commensal dysbiosis is a host-intrinsic regulator of tissue inflammation and tumor cell dissemination in hormone receptor-positive breast cancer. Cancer Res..

[25] McKee A.M., Kirkup B.M., Madgwick M., Fowler W.J., Price C.A., Dreger S.A., Ansorge R., Makin K.A., Caim S., Le Gall G. (2021). Antibiotic-induced disturbances of the gut microbiota result in accelerated breast tumor growth. iScience.

[26] Rao V.P., Poutahidis T., Ge Z., Nambiar P.R., Boussahmain C., Wang Y.Y., Horwitz B.H., Fox J.G., Erdman S.E. (2006). Innate immune inflammatory response against enteric bacteria Helicobacter hepaticus induces mammary adenocarcinoma in mice. Cancer Res..

[27] Kaakoush N.O. (2015). Insights into the role of erysipelotrichaceae in the human host. Front. Cell. Infect. Microbiol..

[28] Sampsell K., Hao D., Reimer R.A. (2020). The gut microbiota: A potential gateway to improved health outcomes in breast cancer treatment and survivorship. Int. J. Mol. Sci..

[29] Miko E., Kovacs T., Sebo E., Toth J., Csonka T., Ujlaki G., Sipos A., Szabó J., Méhes G., Bai P. (2019). Microbiome-microbial metabolome-cancer cell interactions in breast cancer-familiar, but unexplored. Cells.

[30] Horigome A., Okubo R., Hamazaki K., Kinoshita T., Katsumata N., Uezono Y., Xiao J., Matsuoka Y. (2019). Association between blood omega-3 polyunsaturated fatty acids and the gut microbiota among breast cancer survivors. Benef. Microbes.

[31] LeMay-Nedjelski L., Mason-Ennis J.K., Taibi A., Comelli E.M., Thompson L.U. (2018). Omega-3 polyunsaturated fatty acids time-dependently reduce cell viability and oncogenic MicroRNA-21 expression in estrogen receptor-positive breast cancer cells (MCF-7). Int. J. Mol. Sci..

[32] Oliphant K., Allen-Vercoe E. (2019). Macronutrient metabolism by the human gut microbiome: Major fermentation by-products and their impact on host health. Microbiome.

[33] Chuah L.-O., Foo H.L., Loh T.C., Alitheen N.B.M., Yeap S.K., Mutalib N.E.A., Rahim R.A., Yusoff K. (2019). Postbiotic metabolites produced by *Lactobacillus plantarum* strains exert selective cytotoxicity effects on cancer cells. BMC Complement. Altern. Med..

[34] Pessione E. (2012). Lactic acid bacteria contribution to gut microbiota complexity: Lights and shadows. Front. Cell. Infect. Microbiol..

[35] Wang L.S., Huang Y.W., Liu S., Yan P., Lin Y.C. (2008). Conjugated linoleic acid induces apoptosis through estrogen receptor alpha in human breast tissue. BMC Cancer.

[36] Kemp M.Q., Jeffy B.D., Romagnolo D.F. (2003). Conjugated linoleic acid inhibits cell proliferation through a p53-dependent mechanism: Effects on the expression of G1-restriction points in breast and colon cancer cells. J. Nutr..

[37] Parada Venegas D., De la Fuente M.K., Landskron G., Gonzalez M.J., Quera R., Dijkstra G., Harmsen H.J.M., Faber K.N., Hermoso M.A. (2019). Short chain fatty acids (SCFAs)-mediated gut epithelial and immune regulation and its relevance for inflammatory bowel diseases. Front. Immunol..

[38] Park H.S., Han J.H., Park J.W., Lee D.H., Jang K.W., Lee M., Heo K.S., Myung C.-S. (2021). Sodium propionate exerts anticancer effect in mice bearing breast cancer cell xenograft by regulating JAK2/STAT3/ROS/p38 MAPK signaling. Acta Pharmacol. Sin..

[39] Al-Ansari M.M., AlMalki R.H., Dahabiyeh L.A., Abdel Rahman A.M. (2021). Metabolomics-microbiome crosstalk in the breast cancer microenvironment. Metabolites.

[40] Gu X., Chen J., Li H., Song Z., Chang L., He X., Fan Z. (2021). Isomaltooligosaccharide and Bacillus regulate the duration of farrowing and weaning-estrous interval in sows during the perinatal period by changing the gut microbiota of sows. Anim. Nutr..

[41] Ma J., Sun L., Liu Y., Ren H., Shen Y., Bi F., Zhang T., Wang X. (2020). Alter between gut bacteria and blood metabolites and the anti-tumor effects of Faecalibacterium prausnitzii in breast cancer. BMC Microbiol..

[42] Hosseini E., Grootaert C., Verstraete W., Van de Wiele T. (2011). Propionate as a health-promoting microbial metabolite in the human gut. Nutr. Rev..

[43] Mirzaei R., Afaghi A., Babakhani S., Sohrabi M.R., Hosseini-Fard S.R., Babolhavaeji K., Akbari S.K.A., Yousefimashouf R., Karampoor S. (2021). Role of microbiota-derived short-chain fatty acids in cancer development and prevention. Biomed. Pharmacother..

[44] Wang W., Nag S.A., Zhang R. (2015). Targeting the NFkappaB signaling pathways for breast cancer prevention and therapy. Curr. Med. Chem..

[45] Shi F., Li Y., Han R., Fu A., Wang R., Nusbaum O., Qin Q., Chen X., Hou L., Zhu Y. (2021). Valerian and valeric acid inhibit growth of breast cancer cells possibly by mediating epigenetic modifications. Sci. Rep..

[46] Wilson A.S., Koller K.R., Ramaboli M.C., Nesengani L.T., Ocvirk S., Chen C., Flanagan C.A., Sapp F.R., Merritt Z.T., Bhatti F. (2020). Diet and the human gut microbiome: An international review. Dig. Dis. Sci..

[47] Garmpis N., Damaskos C., Garmpi A., Kalampokas E., Kalampokas T., Spartalis E., Daskalopoulou A., Valsami S., Kontos M., Nonni A. (2017). Histone deacetylases as new therapeutic targets in triple-negative breast cancer: Progress and promises. Cancer Genom. Proteom..

[48] An J., Bin Kim J., Yang E.Y., Kim H.O., Lee W.-H., Yang J., Kwon H., Paik N.S., Lim W., Kim Y.-K. (2021). Bacterial extracellular vesicles affect endocrine therapy in MCF7 cells. Medicine.

[49] Di Modica M., Gargari G., Regondi V., Bonizzi A., Arioli S., Belmonte B., de Cecco L., Fasano E., Bianchi F., Bertolotti A. (2021). Gut microbiota condition the therapeutic efficacy of trastuzumab in HER2-positive breast cancer. Cancer Res..

[50] Juan Z., Chen J., Ding B., Yongping L., Liu K., Wang L., Le Y., Liao Q., Shi J., Huang J. (2022). Probiotic supplement attenuates chemotherapy-related cognitive impairment in patients with breast cancer: A randomised, double-blind, and placebo-controlled trial. Eur. J. Cancer.

[51] Fruge A.D., Van der Pol W., Rogers L.Q., Morrow C.D., Tsuruta Y., Demark-Wahnefried W. (2020). Fecal Akkermansia muciniphila is associated with body composition and microbiota diversity in overweight and obese women with breast cancer participating in a presurgical weight loss trial. J. Acad. Nutr. Diet..

[52] Parida S., Wu S., Siddharth S., Wang G., Muniraj N., Nagalingam A., Hum C., Mistriotis P., Hao H., Talbot C.C. (2021). A procarcinogenic colon microbe promotes breast tumorigenesis and metastatic progression and concomitantly activates notch and beta-catenin axes. Cancer Discov..

[53] Shi J., Geng C., Sang M., Gao W., Li S., Yang S., Li Z. (2019). Effect of gastrointestinal microbiome and its diversity on the expression of tumor-infiltrating lymphocytes in breast cancer. Oncol Lett..

[54] Zhang J., Xia Y., Sun J. (2021). Breast and gut microbiome in health and cancer. Genes Dis..

[55] Bhatelia K., Singh K., Singh R. (2014). TLRs: Linking inflammation and breast cancer. Cell. Signal..

[56] Lakritz J.R., Poutahidis T., Mirabal S., Varian B.J., Levkovich T., Ibrahim Y.M., Ward J.M., Teng E.C., Fisher B., Parry N. (2015). Gut bacteria require neutrophils to promote mammary tumorigenesis. Oncotarget.

[57] Erdman S.E., Poutahidis T., Tomczak M., Rogers A.B., Cormier K., Plank B., Horwitz B.H., Fox J.G. (2003). CD4+ CD25+ regulatory T lymphocytes inhibit microbially induced colon cancer in Rag2-deficient mice. Am. J. Pathol..

[58] Shastri A.A., Saleh A., Savage J.E., DeAngelis T., Camphausen K., Simone N.L. (2020). Dietary alterations modulate the microRNA 29/30 and IGF-1/AKT signaling axis in breast cancer liver metastasis. Nutr. Metab..

[59] Simone B.A., Dan T., Palagani A., Jin L., Han S.Y., Wright C., Savage J.E., Gitman R., Lim M.K., Palazzo J. (2016). Caloric restriction coupled with radiation decreases metastatic burden in triple negative breast cancer. Cell Cycle.

[60] Hou M.-F., Ou-Yang F., Li C.-L., Chen F.-M., Chuang C.-H., Kan J.-Y., Wu C.-C., Shih S.-L., Shiau J.-P., Kao L.-C. (2021). Comprehensive profiles and diagnostic value of menopausal-specific gut microbiota in premenopausal breast cancer. Exp. Mol. Med..

[61] Zhu J., Liao M., Yao Z., Liang W., Li Q., Liu J., Yang H., Ji Y., Wei W., Tan A. (2018). Breast cancer in postmenopausal women is associated with an altered gut metagenome. Microbiome.

[62] Goedert J.J., Hua X., Bielecka A., Okayasu I., Milne G.L., Jones G.S., Fujiwara M., Sinha R., Wan Y., Xu X. (2018). Postmenopausal breast cancer and oestrogen associations with the IgA-coated and IgA-noncoated faecal microbiota. Br. J. Cancer.

[63] Fuhrman B.J., Schairer C., Gail M.H., Boyd-Morin J., Xu X., Sue L.Y., Buys S.S., Isaacs C., Keefer L.K., Veenstra T.D. (2012). Estrogen metabolism and risk of breast cancer in postmenopausal women. J. Natl. Cancer Inst..

[64] Ervin S.M., Li H., Lim L., Roberts L.R., Liang X., Mani S., Redinbo M.R. (2019). Gut microbial beta-glucuronidases reactivate estrogens as components of the estrobolome that reactivate estrogens. J. Biol. Chem..

[65] Kwa M., Plottel C.S., Blaser M.J., Adams S. (2016). The intestinal microbiome and estrogen receptor-positive female breast cancer. J. Natl. Cancer Inst..

[66] Flores R., Shi J., Fuhrman B., Xu X., Veenstra T.D., Gail M.H., Gajer P., Ravel J., Goedert J.J. (2012). Fecal microbial determinants of fecal and systemic estrogens and estrogen metabolites: A cross-sectional study. J. Transl. Med..

[67] Sui Y., Wu J., Chen J. (2021). The role of gut microbial beta-glucuronidase in estrogen reactivation and breast cancer. Front. Cell. Dev. Biol..

[68] Zengul A.G., Demark-Wahnefried W., Barnes S., Morrow C.D., Bertrand B., Berryhill T.F., Frugé A.D. (2021). Associations between dietary fiber, the fecal microbiota and estrogen metabolism in postmenopausal women with breast cancer. Nutr. Cancer.

[69] Wu A.H., Tseng C., Vigen C., Yu Y., Cozen W., Garcia A.A., Spicer D. (2020). Gut microbiome associations with breast cancer risk factors and tumor characteristics: A pilot study. Breast Cancer Res. Treat..

[70] Parida S., Sharma D. (2019). The microbiome-estrogen connection and breast cancer risk. Cells.

[71] Setchell K.D., Lawson A.M., Borriello S.P., Harkness R., Gordon H., Morgan D.M., Kirk D.N., Adlercreatz H., Anderson L.C., Axelson M. (1981). Lignan formation in man—Microbial involvement and possible roles in relation to cancer. Lancet.

[72] Saarinen N.M., Warri A., Airio M., Smeds A., Makela S. (2007). Role of dietary lignans in the reduction of breast cancer risk. Mol. Nutr. Food Res..

[73] Eslami S.Z., Majidzadeh A.K., Halvaei S., Babapirali F., Esmaeili R. (2020). Microbiome and breast cancer: New role for an ancient population. Front. Oncol..

[74] Falk R.T., Brinton L.A., Dorgan J.F., Fuhrman B.J., Veenstra T.D., Xu X., Gierach G.L. (2013). Relationship of serum estrogens and estrogen metabolites to postmenopausal breast cancer risk: A nested case-control study. Breast Cancer Res..

[75] Fuhrman B., Feigelson H.S., Flores R., Gail M.H., Xu X., Ravel J., Goedert J.J. (2014). Associations of the fecal microbiome with urinary estrogens and estrogen metabolites in postmenopausal women. J. Clin. Endocrinol. Metab..

[76] Teng N.M.Y., Price C.A., McKee A.M., Hall L.J., Robinson S.D. (2021). Exploring the impact of gut microbiota and diet on breast cancer risk and progression. Int. J. Cancer.

[77] Xiao Y., Xia J., Li L., Ke Y., Cheng J., Xie Y., Chu W., Cheung P., Kim J.H., Colditz G.A. (2019). Associations between dietary patterns and the risk of breast cancer: A systematic review and meta-analysis of observational studies. Breast Cancer Res..

[78] Elhenawy W., Debelyy M.O., Feldman M.F. (2014). Preferential packing of acidic glycosidases and proteases into bacteroides outer membrane vesicles. mBio.

[79] Jin J.S., Touyama M., Hisada T., Benno Y. (2012). Effects of green tea consumption on human fecal microbiota with special reference to bifidobacterium species. Microbiol. Immunol..

[80] Hieken T.J., Chen J., Hoskin T.L., Walther-Antonio M., Johnson S., Ramaker S., Xiao J., Radisky D.C., Knutson K.L., Kalari K. (2016). The microbiome of aseptically collected human breast tissue in benign and malignant disease. Sci. Rep..

[81] Fernandez L., Pannaraj P.S., Rautava S., Rodriguez J.M. (2020). The microbiota of the human mammary ecosystem. Front. Cell. Infect. Microbiol..

[82] Collado M.C., Rautava S., Aakko J., Isolauri E., Salminen S. (2016). Human gut colonisation may be initiated in utero by distinct microbial communities in the placenta and amniotic fluid. Sci. Rep..

[83] Rescigno M., Urbano M., Valzasina B., Francolini M., Rotta G., Bonasio R., Granucci F., Kraehenbuhl J.-P., Ricciardi-Castagnoli P. (2001). Dendritic cells express tight junction proteins and penetrate gut epithelial monolayers to sample bacteria. Nat. Immunol..

[84] Angelopoulou A., Field D., Ryan C.A., Stanton C., Hill C., Ross R.P. (2018). The microbiology and treatment of human mastitis. Med. Microbiol. Immunol..

[85] Adiliaghdam F., Almpani M., Gharedaghi M.H., Najibi M., Hodin R.A., Rahme L.G. (2019). Targeting bacterial quorum sensing shows promise in improving intestinal barrier function following burnsite infection. Mol. Med. Rep..

[86] Asnicar F., Manara S., Zolfo M., Truong D.T., Scholz M., Armanini F., Ferretti P., Gorfer V., Pedrotti A., Tett A. (2017). Studying vertical microbiome transmission from mothers to infants by strain-level metagenomic profiling. mSystems.

[87] Ojo-Okunola A., Nicol M., du Toit E. (2018). Human breast milk bacteriome in health and disease. Nutrients.

[88] Kovacs T., Miko E., Ujlaki G., Sari Z., Bai P. (2020). The microbiome as a component of the tumor microenvironment. Adv. Exp. Med. Biol..

[89] Zhang J., Lu R., Zhang Y., Matuszek Ż, Zhang W., Xia Y., Pan T., Sun J. (2020). tRNA queuosine modification enzyme modulates the growth and microbiome recruitment to breast tumors. Cancers.

[90] Urbaniak C., Cummins J., Brackstone M., Macklaim J.M., Gloor G.B., Baban C.K., Scott L., O’Hanlon D.M., Burton J.P., Francis K.P. (2014). Microbiota of human breast tissue. Appl. Environ. Microbiol..

[91] Haque S., Raina R., Afroze N., Hussain A., Alsulimani A., Singh V., Mishra B.N., Kaul S., Kharwar R.N. (2021). Microbial dysbiosis and epigenetics modulation in cancer development—A chemopreventive approach. Semin. Cancer Biol..

[92] Soto-Pantoja D.R., Gaber M., Arnone A.A., Bronson S.M., Cruz-Diaz N., Wilson A.S., Clear K.Y.J., Ramirez M.U., Kucera G.L., Levine E.A. (2021). Diet alters entero-mammary signaling to regulate the breast microbiome and tumorigenesis. Cancer Res..

[93] Thompson K.J., Ingle J.N., Tang X., Chia N., Jeraldo P.R., Walther-Antonio M., Kandimalla K.K., Johnson S., Yao J.Z., Harrington J. (2017). A comprehensive analysis of breast cancer microbiota and host gene expression. PLoS ONE.

[94] Esposito M.V., Fosso B., Nunziato M., Casaburi G., D’Argenio V., Calabrese A., D’Aiuto M., Botti G., Pesole G., Salvatore F. (2022). Microbiome composition indicate dysbiosis and lower richness in tumor breast tissues compared to healthy adjacent paired tissue, within the same women. BMC Cancer.

[95] Meng S., Chen B., Yang J., Wang J., Zhu D., Meng Q., Zhang L. (2018). Study of microbiomes in aseptically collected samples of human breast tissue using needle biopsy and the potential role of in situ tissue microbiomes for promoting malignancy. Front. Oncol..

[96] Urbaniak C., Gloor G.B., Brackstone M., Scott L., Tangney M., Reid G. (2016). The microbiota of breast tissue and its association with breast cancer. Appl. Environ. Microbiol..

[97] Wang N., Sun T., Xu J. (2021). Tumor-related microbiome in the breast microenvironment and breast cancer. J. Cancer.

[98] Smith A., Pierre J.F., Makowski L., Tolley E., Lyn-Cook B., Lu L., Vidal G., Starlard-Davenport A. (2019). Distinct microbial communities that differ by race, stage, or breast-tumor subtype in breast tissues of non-Hispanic Black and non-Hispanic White women. Sci. Rep..

[99] Banerjee S., Wei Z., Tan F., Peck K.N., Shih N., Feldman M., Rebbeck T.R., Alwine J.C., Robertson E.S. (2015). Distinct microbiological signatures associated with triple negative breast cancer. Sci. Rep..

[100] Saud Hussein A., Ibraheem Salih N., Hashim Saadoon I. (2021). Effect of microbiota in the development of breast cancer. Arch. Razi Inst..

[101] Murphy K., Curley D., O’Callaghan T., O’Shea C.-A., Dempsey E.M., O’Toole P., Ross R., Ryan C.A., Stanton C. (2017). The composition of human milk and infant faecal microbiota over the first three months of life: A pilot study. Sci. Rep..

[102] James K., Bottacini F., Contreras J.I.S., Vigoureux M., Egan M., Motherway M.O., Holmes E., Van Sinderen D. (2020). Author correction: Metabolism of the predominant human milk oligosaccharide fucosyllactose by an infant gut commensal. Sci. Rep..

[103] Donnet-Hughes A., Perez P.F., Doré J., Leclerc M., Levenez F., Benyacoub J., Serrant P., Segura-Roggero I., Schiffrin E.J. (2010). Potential role of the intestinal microbiota of the mother in neonatal immune education. Proc. Nutr. Soc..

[104] Babakobi M., Reshef L., Gihaz S., Belgorodsky B., Fishman A., Bujanover Y., Gophna U. (2020). Effect of maternal diet and milk lipid composition on the infant gut and maternal milk microbiomes. Nutrients.

[105] Li M., Bai Y., Zhou J., Huang W., Yan J., Tao J., Fan Q., Liu Y., Mei D., Yan Q. (2019). Core fucosylation of maternal milk N-glycan evokes B cell activation by selectively promoting the l-fucose metabolism of gut *Bifidobacterium* spp. and *Lactobacillus* spp.. mBio.

[106] Bäckhed F., Roswall J., Peng Y., Feng Q., Jia H., Kovatcheva-Datchary P., Li Y., Xia Y., Xie H., Zhong H. (2015). Dynamics and stabilization of the human gut microbiome during the first year of life. Cell Host Microbe.

[107] Thongaram T., Hoeflinger J.L., Chow J., Miller M.J. (2017). Human milk oligosaccharide consumption by probiotic and human-associated bifidobacteria and lactobacilli. J. Dairy Sci..

[108] Pichler M.J., Yamada C., Shuoker B., Alvarez-Silva C., Gotoh A., Leth M.L., Schoof E., Katoh T., Sakanaka M., Katayama T. (2020). Butyrate producing colonic Clostridiales metabolise human milk oligosaccharides and cross feed on mucin via conserved pathways. Nat. Commun..

[109] Stinson L., Gay M.C.L., Koleva P.T., Eggesbø M., Johnson C.C., Wegienka G., Du Toit E., Shimojo N., Munblit D., Campbell D.E. (2020). Human milk from atopic mothers has lower levels of short chain fatty acids. Front. Immunol..

[110] Maldonado J., Lara-Villoslada F., Sierra S., Sempere L., Gómez M., Rodriguez J.M., Boza J., Xaus J., Olivares M. (2010). Safety and tolerance of the human milk probiotic strain Lactobacillus salivarius CECT5713 in 6-month-old children. Nutrition.

[111] Bouladoux N., Hall J.A., Grainger J.R., dos Santos L.M., Kann M.G., Nagarajan V., Nagarajan V., Verthelyi D., Belkaid Y. (2012). Regulatory role of suppressive motifs from commensal DNA. Mucosal Immunol..

[112] Ward T.L., Hosid S., Ioshikhes I., Altosaar I. (2013). Human milk metagenome: A functional capacity analysis. BMC Microbiol..

[113] Mastromarino P., Capobianco D., Miccheli A., Praticò G., Campagna G., Laforgia N., Capursi T., Baldassarre M.E. (2015). Administration of a multistrain probiotic product (VSL#3) to women in the perinatal period differentially affects breast milk beneficial microbiota in relation to mode of delivery. Pharmacol. Res..

[114] Laborda-Illanes A., Sanchez-Alcoholado L., Dominguez-Recio M.E., Jimenez-Rodriguez B., Lavado R., Comino-Méndez I., Alba E., Queipo-Ortuño M.I. (2020). Breast and gut microbiota action mechanisms in breast cancer pathogenesis and treatment. Cancers.

[115] Dorgan J.F., Baer D.J., Albert P.S., Judd J.T., Brown E.D., Corle N.K., Campbell W.S., Hartman T.J., Tejpar A.A., Clevidence B.A. (2001). Serum hormones and the alcohol-breast cancer association in postmenopausal women. J. Natl. Cancer Inst..

[116] Subbaramaiah K., Morris P.G., Zhou X.K., Morrow M., Du B., Giri D., Kopelovich L., Hudis C.A., Dannenberg A.J. (2012). Increased levels of COX-2 and prostaglandin E2 contribute to elevated aromatase expression in inflamed breast tissue of obese women. Cancer Discov..

[117] Vergara D., Simeone P., Damato M., Maffia M., Lanuti P., Trerotola M. (2019). The cancer microbiota: EMT and inflammation as shared molecular mechanisms associated with plasticity and progression. J. Oncol..

[118] Kovács T., Mikó E., Vida A., Sebő É., Toth J., Csonka T., Boratkó A., Ujlaki G., Lente G., Kovács P. (2019). Cadaverine, a metabolite of the microbiome, reduces breast cancer aggressiveness through trace amino acid receptors. Sci. Rep..

[119] Shively C.A., Register T.C., Appt S.E., Clarkson T.B., Uberseder B., Clear K.Y., Wilson A.S., Chiba A., Tooze J.A., Cook K.L. (2018). Consumption of mediterranean versus western diet leads to distinct mammary gland microbiome populations. Cell Rep..

[120] Mikó E., Vida A., Kovács T., Ujlaki G., Trencsényi G., Márton J., Sári Z., Kovács P., Boratkó A., Hujber Z. (2018). Lithocholic acid, a bacterial metabolite reduces breast cancer cell proliferation and aggressiveness. Biochim. Biophys. Acta Bioenerg..

[121] Lindahl G., Abrahamsson A., Dabrosin C. (2019). Dietary flaxseed and tamoxifen affect the inflammatory microenvironment in vivo in normal human breast tissue of postmenopausal women. Eur. J. Clin. Nutr..

[122] Viola G.M., Baumann D.P., Mohan K., Selber J., Garvey P., Reece G., Raad I.I., Rolston K.V., Crosby M.A. (2016). Improving antimicrobial regimens for the treatment of breast tissue expander-related infections. Plast. Reconstr. Surg. Glob. Open.

[123] Chiba A., Bawaneh A., Velazquez C., Clear K.Y., Wilson A.S., Howard-McNatt M., Levine E.A., Levi-Polyachenko N., Yates-Alston S.A., Diggle S.P. (2020). Neoadjuvant chemotherapy shifts breast tumor microbiota populations to regulate drug responsiveness and the development of metastasis. Mol. Cancer Res..

[124] Dieleman S., Aarnoutse R., Ziemons J., Kooreman L., Boleij A., Smidt M. (2021). Exploring the potential of breast microbiota as biomarker for breast cancer and therapeutic response. Am. J. Pathol..

[125] Sedighi M., Bialvaei A.Z., Hamblin M.R., Ohadi E., Asadi A., Halajzadeh M., Lohrasbi V., Mohammadzadeh N., Amiriani T., Krutova M. (2019). Therapeutic bacteria to combat cancer; current advances, challenges, and opportunities. Cancer Med..

[126] Lertpiriyapong K., Whary M.T., Muthupalani S., Lofgren J.L., Gamazon E.R., Feng Y., Ge Z., Wang T.C., Fox J.G. (2014). Gastric colonisation with a restricted commensal microbiota replicates the promotion of neoplastic lesions by diverse intestinal microbiota in the Helicobacter pylori INS-GAS mouse model of gastric carcinogenesis. Gut.

[127] Pellegrini M., Ippolito M., Monge T., Violi R., Cappello P., Ferrocino I., Cocolin L.S., De Francesco A., Bo S., Finocchiaro C. (2020). Gut microbiota composition after diet and probiotics in overweight breast cancer survivors: A randomized open-label pilot intervention trial. Nutrition.

[128] Xu H., Hiraishi K., Kurahara L.-H., Nakano-Narusawa Y., Li X., Hu Y., Matsuda Y., Zhang H., Hirano K. (2021). Inhibitory effects of breast milk-derived lactobacillus rhamnosus probio-M9 on colitis-associated carcinogenesis by restoration of the gut microbiota in a mouse model. Nutrients.

[129] Gorska A., Przystupski D., Niemczura M.J., Kulbacka J. (2019). Probiotic bacteria: A promising tool in cancer prevention and therapy. Curr. Microbiol..

[130] Lakritz J.R., Poutahidis T., Levkovich T., Varian B.J., Ibrahim Y.M., Chatzigiagkos A., Mirabal S., Alm E.J., Erdman S.E. (2014). Beneficial bacteria stimulate host immune cells to counteract dietary and genetic predisposition to mammary cancer in mice. Int. J. Cancer.

[131] Yazdi M.H., Soltan Dallal M.M., Hassan Z.M., Holakuyee M., Agha Amiri S., Abolhassani M., Mahdavi M. (2010). Oral administration of *Lactobacillus acidophilus* induces IL-12 production in spleen cell culture of BALB/c mice bearing transplanted breast tumour. Br. J. Nutr..

[132] de Moreno de LeBlanc A., Matar C., Theriault C., Perdigon G. (2005). Effects of milk fermented by Lactobacillus helveticus R389 on immune cells associated to mammary glands in normal and a breast cancer model. Immunobiology.

[133] Fessler J., Matson V., Gajewski T.F. (2019). Exploring the emerging role of the microbiome in cancer immunotherapy. J. Immunother. Cancer.

[134] Strouse C., Mangalam A., Zhang J. (2019). Bugs in the system: Bringing the human microbiome to bear in cancer immunotherapy. Gut Microbes.

[135] Sivan A., Corrales L., Hubert N., Williams J.B., Aquino-Michaels K., Earley Z.M., Benyamin F.W., Lei Y.M., Jabri B., Alegre M.-L. (2015). Commensal Bifidobacterium promotes antitumor immunity and facilitates anti-PD-L1 efficacy. Science.

[136] Kim E., Ahn H., Park H. (2021). A review on the role of gut microbiota in immune checkpoint blockade therapy for cancer. Mamm. Genome.

[137] Vivarelli S., Salemi R., Candido S., Falzone L., Santagati M., Stefani S., Torino F., Banna G.L., Tonini G., Libra M. (2019). Gut microbiota and cancer: From pathogenesis to therapy. Cancers.

[138] Vétizou M., Pitt J.M., Daillère R., Lepage P., Waldschmitt N., Flament C., Rusakiewicz S., Routy B., Roberti M.P., Duong C.P.M. (2015). Anticancer immunotherapy by CTLA-4 blockade relies on the gut microbiota. Science.

[139] Singh A., Nayak N., Rathi P., Verma D., Sharma R., Chaudhary A., Agarwal A., Tripathi Y.B., Garg N. (2021). Microbiome and host crosstalk: A new paradigm to cancer therapy. Semin. Cancer Biol..

[140] Yonezawa T., Kobayashi Y., Obara Y. (2007). Short-chain fatty acids induce acute phosphorylation of the p38 mitogen-activated protein kinase/heat shock protein 27 pathway via GPR43 in the MCF-7 human breast cancer cell line. Cell Signal..

[141] Nakkarach A., Foo H.L., Song A.A., Mutalib N.E.A., Nitisinprasert S., Withayagiat U. (2021). Anti-cancer and anti-inflammatory effects elicited by short chain fatty acids produced by *Escherichia coli* isolated from healthy human gut microbiota. Microb. Cell Factories.

[142] Johnson D.E., O’Keefe R.A., Grandis J.R. (2018). Targeting the IL-6/JAK/STAT3 signalling axis in cancer. Nat. Rev. Clin. Oncol..

[143] Hassan Z., Mustafa S., Rahim R.A., Isa N.M. (2016). Anti-breast cancer effects of live, heat-killed and cytoplasmic fractions of *Enterococcus faecalis* and *Staphylococcus hominis* isolated from human breast milk. Vitr. Cell. Dev. Biol. Anim..

[144] Bobin-Dubigeon C., Bard J.M., Luu T.H., Le Vacon F., Nazih H. (2020). Basolateral secretion from Caco-2 cells pretreated with fecal waters from breast cancer patients affects MCF7 cell viability. Nutrients.

[145] Alcon-Giner C., Dalby M.J., Caim S., Ketskemety J., Shaw A., Sim K., Lawson M.A., Kiu R., LeClaire C., Chalklen L. (2020). Microbiota supplementation with bifidobacterium and lactobacillus modifies the preterm infant gut microbiota and metabolome: An observational study. Cell Rep. Med..

[146] Toi M., Hirota S., Tomotaki A., Sato N., Hozumi Y., Anan K., Nagashima T., Tokuda Y., Masuda N., Ohsumi S. (2013). Probiotic beverage with soy isoflavone consumption for breast cancer prevention: A case-control study. Curr. Nutr. Food Sci..

[147] Waters C.M., Bassler B.L. (2005). Quorum sensing: Cell-to-cell communication in bacteria. Annu. Rev. Cell. Dev. Biol..

[148] De Spiegeleer B., Verbeke F., D’Hondt M., Hendrix A., Van De Wiele C., Burvenich C., Peremans K., de Wever O., Bracke M., Wynendaele E. (2015). The quorum sensing peptides PhrG, CSP and EDF promote angiogenesis and invasion of breast cancer cells in vitro. PLoS ONE.

[149] Tornesello A.L., Buonaguro L., Tornesello M.L., Buonaguro F.M. (2018). The role of sensing peptides in the cross-talk between microbiota and human cancer cells. Mini Rev. Med. Chem..

[150] Neuzillet C., Tijeras-Raballand A., Cohen R., Cros J., Faivre S., Raymond E., de Gramont A. (2015). Targeting the TGFbeta pathway for cancer therapy. Pharmacol. Ther..

[151] Shi L., Sheng J., Wang M., Luo H., Zhu J., Zhang B., Liu Z., Yang X. (2019). Combination therapy of TGF-beta blockade and commensal-derived probiotics provides enhanced antitumor immune response and tumor suppression. Theranostics.

[152] Sun H., Chen Y., Cheng M., Zhang X., Zheng X., Zhang Z. (2018). The modulatory effect of polyphenols from green tea, oolong tea and black tea on human intestinal microbiota in vitro. J. Food Sci. Technol..

[153] Debras C., Chazelas E., Srour B., Julia C., Schneider E., Kesse-Guyot E., Agaësse C., Druesne-Pecollo N., Andreeva V.A., Wendeu-Foyet G. (2022). Fermentable oligosaccharides, disaccharides, monosaccharides, and polyols (FODMAPs) and cancer risk in the prospective nutrinet-sante cohort. J. Nutr..

[154] Tosti V., Bertozzi B., Fontana L. (2018). Health benefits of the mediterranean diet: Metabolic and molecular mechanisms. J. Gerontol. A Biol. Sci. Med. Sci..

[155] Newman T.M., Vitolins M.Z., Cook K.L. (2019). From the table to the tumor: The role of mediterranean and western dietary patterns in shifting microbial-mediated signaling to impact breast cancer risk. Nutrients.

[156] Han B., Jiang P., Jiang L., Li X., Ye X. (2021). Three phytosterols from sweet potato inhibit MCF7-xenograft-tumor growth through modulating gut microbiota homeostasis and SCFAs secretion. Food Res. Int..

[157] Paul B., Royston K.J., Li Y., Stoll M.L., Skibola C.F., Wilson L.S., Barnes S., Morrow C.D., Tollefsbol T.O. (2017). Impact of genistein on the gut microbiome of humanized mice and its role in breast tumor inhibition. PLoS ONE.

[158] Andrade F.D.O., Liu F., Zhang X., Rosim M.P., Dani C., Cruz I., Wang T.T.Y., Helferich W., Li R.W., Hilakivi-Clarke L. (2021). Genistein reduces the risk of local mammary cancer recurrence and ameliorates alterations in the gut microbiota in the offspring of obese dams. Nutrients.

[159] Teixeira L.L., Costa G.R., Dörr F.A., Ong T.P., Pinto E., Lajolo F.M., Hassimotto N.M.A. (2017). Potential antiproliferative activity of polyphenol metabolites against human breast cancer cells and their urine excretion pattern in healthy subjects following acute intake of a polyphenol-rich juice of grumixama (*Eugenia brasiliensis* Lam.). Food Funct..

[160] Steiner J., Davis J., McClellan J., Enos R., Carson J., Fayad R., Nagarkatti M., Nagarkatti P.S., Altomare D., Creek K.E. (2014). Dose-dependent benefits of quercetin on tumorigenesis in the C3(1)/SV40Tag transgenic mouse model of breast cancer. Cancer Biol. Ther..

[161] Castillo-Pichardo L., Martinez-Montemayor M.M., Martinez J.E., Wall K.M., Cubano L.A., Dharmawardhane S. (2009). Inhibition of mammary tumor growth and metastases to bone and liver by dietary grape polyphenols. Clin. Exp. Metastasis.

[162] Song H., Jung J.I., Cho H.J., Her S., Kwon S.-H., Yu R., Kang Y.-H., Lee K.W., Park J.H.Y. (2015). Inhibition of tumor progression by oral piceatannol in mouse 4T1 mammary cancer is associated with decreased angiogenesis and macrophage infiltration. J. Nutr. Biochem..

[163] Barbieri A., Quagliariello V., Del Vecchio V., Falco M., Luciano A., Amruthraj N.J., Nasti G., Ottaiano A., Berretta M., Iaffaioli R.V. (2017). Anticancer and anti-inflammatory properties of *Ganoderma lucidum* extract effects on melanoma and triple-negative breast cancer treatment. Nutrients.

[164] Su J., Su L., Li D., Shuai O., Zhang Y., Liang H., Jiao C., Xu Z., Lai Y., Xie Y. (2018). Antitumor activity of extract from the sporoderm-breaking spore of *Ganoderma lucidum* : Restoration on exhausted cytotoxic t cell with gut microbiota remodeling. Front. Immunol..

[165] Su J., Li D., Chen Q., Li M., Su L., Luo T., Liang D., Lai G., Shuai O., Jiao C. (2018). Anti-breast cancer enhancement of a polysaccharide from spore of *Ganoderma lucidum* with paclitaxel: Suppression on tumor metabolism with gut microbiota reshaping. Front. Microbiol..

[166] Peterson C.T., Vaughn A.R., Sharma V., Chopra D., Mills P.J., Peterson S.N., Sivamani R.K. (2018). Effects of turmeric and curcumin dietary supplementation on human gut microbiota: A double-blind, randomized, placebo-controlled pilot study. J. Evid. Based Integr. Med..

[167] Tripathi A.K., Ray A.K., Mishra S.K. (2022). Molecular and pharmacological aspects of piperine as a potential molecule for disease prevention and management: Evidence from clinical trials. Beni-Suef Univ. J. Basic Appl. Sci..

[168] Luo D., Hou D., Wen T., Feng M., Zhang H. (2020). Efficacy and safety of Brucea javanica oil emulsion for liver cancer: A protocol for systematic review and meta-analysis. Medicine.

[169] Wu J.R., Liu S.Y., Zhu J.L., Zhang D., Wang K.H. (2018). Efficacy of Brucea javanica oil emulsion injection combined with the chemotherapy for treating gastric cancer: A systematic review and meta-analysis. Evid. Based Complement Alternat. Med..

[170] Su J., Chen X., Xiao Y., Li D., Li M., Li H., Huang J., Lai Z., Su Z., Xie Y. (2021). Bruceae fructus oil inhibits triple-negative breast cancer by restraining autophagy: Dependence on the gut microbiota-mediated amino acid regulation. Front. Pharmacol..

[171] Kim J.-K., Choi M.S., Kim J.-Y., Yu J.S., Seo J.I., Yoo H.H., Kim D.-H. (2021). *Ginkgo biloba* leaf extract suppresses intestinal human breast cancer resistance protein expression in mice: Correlation with gut microbiota. Biomed. Pharmacother..

[172] Xue M., Ji X., Liang H., Liu Y., Wang B., Sun L., Li W. (2018). The effect of fucoidan on intestinal flora and intestinal barrier function in rats with breast cancer. Food Funct..

[173] Siriwardhana N., Kalupahana N.S., Moustaid-Moussa N. (2012). Health benefits of n-3 polyunsaturated fatty acids: Eicosapentaenoic acid and docosahexaenoic acid. Adv. Food Nutr. Res..

[174] Vijay A., Astbury S., Le Roy C., Spector T.D., Valdes A.M. (2021). The prebiotic effects of omega-3 fatty acid supplementation: A six-week randomised intervention trial. Gut Microbes.

[175] Li J., Wan Y., Zheng Z., Zhang H., Li Y., Guo X., Li K., Li D. (2021). Maternal n-3 polyunsaturated fatty acids restructure gut microbiota of offspring mice and decrease their susceptibility to mammary gland cancer. Food Funct..

[176] Al-Shabanah O.A., Alotaibi M.R., Al Rejaie S.S., Alhoshani A.R., Almutairi M.M., Alshammari M.A., Hafez M.M. (2016). Inhibitory effect of ginseng on breast cancer cell line growth via up-regulation of cyclin dependent kinase inhibitor, p21 and p53. Asian Pac. J. Cancer Prev..

[177] Li Z., Ji G.E. (2018). Ginseng and obesity. J. Ginseng Res..

[178] Chen Z., Zhang Z., Liu J., Qi H., Li J., Chen J., Huang Q., Liu Q., Mi J., Li X. (2022). Gut microbiota: Therapeutic targets of ginseng against multiple disorders and ginsenoside transformation. Front. Cell. Infect. Microbiol..

[179] Huang J., Liu D., Wang Y., Liu L., Li J., Yuan J., Jiang Z., Jiang Z., Hsiao W.W., Liu H. (2022). Ginseng polysaccharides alter the gut microbiota and kynurenine/tryptophan ratio, potentiating the antitumour effect of antiprogrammed cell death 1/programmed cell death ligand 1 (anti-PD-1/PD-L1) immunotherapy. Gut.

[180] Meng J., Meng Y., Liang Z., Du L., Zhang Z., Hu X., Shan F. (2013). Phenotypic and functional analysis of the modification of murine bone marrow dendritic cells (BMDCs) induced by neutral Ginseng polysaccharides (NGP). Hum. Vaccines Immunother..

[181] Wang Y., Huang M., Sun R., Pan L. (2015). Extraction, characterization of a Ginseng fruits polysaccharide and its immune modulating activities in rats with Lewis lung carcinoma. Carbohydr. Polym..

[182] Li M., Wang X., Wang Y., Bao S., Chang Q., Liu L., Zhang S., Sun L. (2021). Strategies for remodeling the tumor microenvironment using active ingredients of ginseng—A promising approach for cancer therapy. Front. Pharmacol..

[183] Jiang Y., Fan L. (2021). The effect of Poria cocos ethanol extract on the intestinal barrier function and intestinal microbiota in mice with breast cancer. J. Ethnopharmacol..

[184] Meyer D., Stasse-Wolthuis M. (2009). The bifidogenic effect of inulin and oligofructose and its consequences for gut health. Eur. J. Clin. Nutr..

[185] Miao M., Dai Y., Rui C., Fan Y., Wang X., Fan C., Mu J., Hou W., Dong Z., Li P. (2021). Dietary supplementation of inulin alleviates metabolism disorders in gestational diabetes mellitus mice via RENT/AKT/IRS/GLUT4 pathway. Diabetol. Metab. Syndr..

[186] Singh A., Zapata R.C., Pezeshki A., Reidelberger R.D., Chelikani P.K. (2018). Inulin fiber dose-dependently modulates energy balance, glucose tolerance, gut microbiota, hormones and diet preference in high-fat-fed male rats. J. Nutr. Biochem..

[187] Taper H.S., Roberfroid M.B. (2005). Possible adjuvant cancer therapy by two prebiotics—Inulin or oligofructose. In Vivo.

[188] Kondegowda N.G., Meaney M.P., Baker C., Ju Y.H. (2011). Effects of non-digestible carbohydrates on the growth of estrogen-dependent human breast cancer (MCF-7) tumors implanted in ovariectomized athymic mice. Nutr. Cancer.

[189] Kassayová M., Bobrov N., Strojný L., Kisková T., Mikes J., Demečková V., Orendáš P., Bojková B., Péč M., Kubatka P. (2014). Preventive effects of probiotic bacteria *Lactobacillus plantarum* and dietary fiber in chemically-induced mammary carcinogenesis. Anticancer Res..

[190] Kassayová M., Bobrov N., Strojný L., Orendáš P., Demečková V., Jendželovský R., Kubatka P., Kisková T., Kružliak P., Adamkov M. (2016). Anticancer and immunomodulatory effects of *Lactobacillus plantarum* LS/07, inulin and melatonin in NMU-induced rat model of breast cancer. Anticancer Res..

[191] Sharma M., Arora I., Stoll M.L., Li Y., Morrow C.D., Barnes S., Berryhill T.F., Li S., Tollefsbol T.O. (2020). Nutritional combinatorial impact on the gut microbiota and plasma short-chain fatty acids levels in the prevention of mammary cancer in Her2/neu estrogen receptor-negative transgenic mice. PLoS ONE.

[192] Chen Q., Li Q., Liang Y., Zu M., Chen N., Canup B.S., Luo L., Wang C., Zeng L., Xiao B. (2022). Natural exosome-like nanovesicles from edible tea flowers suppress metastatic breast cancer via ROS generation and microbiota modulation. Acta Pharm. Sin. B.

[193] Luu T.H., Bard J.-M., Carbonnelle D., Chaillou C., Huvelin J.-M., Bobin-Dubigeon C., Nazih H. (2018). Lithocholic bile acid inhibits lipogenesis and induces apoptosis in breast cancer cells. Cell. Oncol..

[194] Rubinstein M.R., Wang X., Liu W., Hao Y., Cai G., Han Y.W. (2013). Fusobacterium nucleatum promotes colorectal carcinogenesis by modulating E-cadherin/beta-catenin signaling via its FadA adhesin. Cell Host Microbe.

[195] Nougayrède J.-P., Homburg S., Taieb F., Boury M., Brzuszkiewicz E., Gottschalk G., Buchrieser C., Hacker J., Dobrindt U., Oswald E. (2006). *Escherichia coli* induces DNA double-strand breaks in eukaryotic cells. Science.

[196] Cuevas-Ramos G., Petit C.R., Marcq I., Boury M., Oswald E., Nougayrede J.P. (2010). *Escherichia coli* induces DNA damage in vivo and triggers genomic instability in mammalian cells. Proc. Natl. Acad. Sci. USA.

[197] Davis C.D., Ross S.A. (2007). Dietary components impact histone modifications and cancer risk. Nutr. Rev..

[198] Hullar M.A., Fu B.C. (2014). Diet, the gut microbiome, and epigenetics. Cancer J..

[199] Okugawa Y., Grady W.M., Goel A. (2015). Epigenetic alterations in colorectal cancer: Emerging biomarkers. Gastroenterology.

[200] Holst B., Williamson G. (2004). A critical review of the bioavailability of glucosinolates and related compounds. Nat. Prod. Rep..

[201] Salimi V., Shahsavari Z., Safizadeh B., Hosseini A., Khademian N., Tavakoli-Yaraki M. (2017). Sodium butyrate promotes apoptosis in breast cancer cells through reactive oxygen species (ROS) formation and mitochondrial impairment. Lipids Health Dis..

[202] Semaan J., El-Hakim S., Ibrahim J.N., Safi R., Elnar A.A., El Boustany C. (2020). Comparative effect of sodium butyrate and sodium propionate on proliferation, cell cycle and apoptosis in human breast cancer cells MCF-7. Breast Cancer.

[203] Greiner A.K., Papineni R.V., Umar S. (2014). Chemoprevention in gastrointestinal physiology and disease. Natural products and microbiome. Am. J. Physiol. Gastrointest. Liver Physiol..

[204] Steed K.L., Jordan H.R., Tollefsbol T.O. (2020). SAHA and EGCG promote apoptosis in triple-negative breast cancer cells, possibly through the modulation of cIAP2. Anticancer Res..

[205] De Figueiredo S.M., Binda N.S., Nogueira-Machado J.A., Vieira-Filho S.A., Caligiorne R.B. (2015). The antioxidant properties of organosulfur compounds (sulforaphane). Recent Pat. Endocr. Metab. Immune Drug Discov..

[206] Meeran S.M., Patel S.N., Tollefsbol T.O. (2010). Sulforaphane causes epigenetic repression of hTERT expression in human breast cancer cell lines. PLoS ONE.

[207] Li S., Chen M., Wu H., Li Y., Tollefsbol T.O. (2020). Maternal epigenetic regulation contributes to prevention of estrogen receptor-negative mammary cancer with broccoli sprout consumption. Cancer Prev. Res..

[208] Li S., Wu H., Tollefsbol T.O. (2021). Combined broccoli sprouts and green tea polyphenols contribute to the prevention of estrogen receptor-negative mammary cancer via cell cycle arrest and inducing apoptosis in HER2/neu mice. J. Nutr..

[209] De Silva S., Tennekoon K.H., Karunanayake E.H. (2021). Interaction of gut microbiome and host microRNAs with the occurrence of colorectal and breast cancer and their impact on patient immunity. OncoTargets Ther..

[210] Carter J.V., Galbraith N.J., Yang D., Burton J.F., Walker S.P., Galandiuk S. (2017). Blood-based microRNAs as biomarkers for the diagnosis of colorectal cancer: A systematic review and meta-analysis. Br. J. Cancer.

[211] Allegra A., Musolino C., Tonacci A., Pioggia G., Gangemi S. (2020). Interactions between the microRNAs and microbiota in cancer development: Roles and therapeutic opportunities. Cancers.

[212] Liu S., da Cunha A.P., Rezende R.M., Cialic R., Wei Z., Bry L., Comstock L.E., Gandhi R., Weiner H.L. (2016). The host shapes the gut microbiota via fecal MicroRNA. Cell Host Microbe.

[213] Wang F., Zheng Z., Guo J., Ding X. (2010). Correlation and quantitation of microRNA aberrant expression in tissues and sera from patients with breast tumor. Gynecol. Oncol..

[214] Yang Y., Alderman C., Sehlaoui A., Xiao Y., Wang W. (2018). MicroRNAs as immunotherapy targets for treating gastroenterological cancers. Can. J. Gastroenterol. Hepatol..

